# The Ability of Lytic Staphylococcal Podovirus vB_SauP_phiAGO1.3 to Coexist in Equilibrium With Its Host Facilitates the Selection of Host Mutants of Attenuated Virulence but Does Not Preclude the Phage Antistaphylococcal Activity in a Nematode Infection Model

**DOI:** 10.3389/fmicb.2018.03227

**Published:** 2019-01-18

**Authors:** Aleksandra Głowacka-Rutkowska, Agnieszka Gozdek, Joanna Empel, Jan Gawor, Karolina Żuchniewicz, Aleksandra Kozińska, Janusz Dębski, Robert Gromadka, Małgorzata Łobocka

**Affiliations:** ^1^Department of Microbial Biochemistry, Institute of Biochemistry and Biophysics, Polish Academy of Sciences, Warsaw, Poland; ^2^Department of Epidemiology and Clinical Microbiology, National Medicines Institute, Warsaw, Poland; ^3^Laboratory of DNA Sequencing and Oligonucleotide Synthesis, Institute of Biochemistry and Biophysics, Polish Academy of Sciences, Warsaw, Poland; ^4^Laboratory of Mass Spectrometry, Institute of Biochemistry and Biophysics, Polish Academy of Sciences, Warsaw, Poland; ^5^Autonomous Department of Microbial Biology, Faculty of Agriculture and Biology, Warsaw University of Life Sciences, Warsaw, Poland

**Keywords:** ArlRS, bacteriophage carrier state, bacteriophage host range, biofilm, *Caenorhabditis elegans* infection model, phage therapy, restriction-modification systems, *Staphylococcus Rosenblumvirus* genus phage

## Abstract

Phage vB_SauP_phiAGO1.3 (phiAGO1.3) is a polyvalent *Staphylococcus* lytic podovirus with a 17.6-kb genome (Gozdek et al., 2018). It can infect most of the *Staphylococcus aureus* human isolates of dominant clonal complexes. We show that a major factor contributing to the wide host range of phiAGO1.3 is a lack or sparcity of target sites for certain restriction-modification systems of types I and II in its genome. Phage phiAGO1.3 requires for adsorption β-*O*-GlcNAcylated cell wall teichoic acid, which is also essential for the expression of methicillin resistance. Under certain conditions an exposure of *S. aureus* to phiAGO1.3 can lead to the establishment of a mixed population in which the bacteria and phages remain in equilibrium over multiple generations. This is reminiscent of the so called phage carrier state enabling the co-existence of phage-resistant and phage-sensitive cells supporting a continuous growth of the bacterial and phage populations. The stable co-existence of bacteria and phage favors the emergence of phage-resistant variants of the bacterium. All phiAGO1.3-resistant cells isolated from the phage-carrier-state cultures contained a mutation inactivating the two-component regulatory system ArlRS, essential for efficient expression of numerous *S. aureus* virulence-associated traits. Moreover, the mutants were unaffected in their susceptibility to infection with an unrelated, polyvalent *S. aureus* phage of the genus *Kayvirus*. The ability of phiAGO1.3 to establish phage-carrier-state cultures did not preclude its antistaphylococcal activity *in vivo* in an *S. aureus* nematode infection model. Taken together our results suggest that phiAGO1.3 could be suitable for the therapeutic application in humans and animals, alone or in cocktails with *Kayvirus* phages. It might be especially useful in the treatment of infections with the majority of methicillin-resistant *S. aureus* strains.

## Introduction

*Staphylococcus aureus* is among the most challenging bacterial pathogens that cause various types of infections mainly in humans but also in animals (Springer et al., 2009; Otto, 2012). It has developed resistance to virtually all categories of antibiotic available for clinical use, but only methicillin-resistant *S. aureus* (MRSA) has reached epidemic proportions globally and has become a major worldwide problem (Mandell et al., 2010). MRSA strains are common both in nosocomial and in community settings (Bal et al., 2016). The limitation of effective MRSA treatment options with antibiotics often leads to the development of chronic infections and is a cause of increased mortality, longer hospital stays and higher health care costs as compared to methicillin-sensitive *S. aureus* (MSSA) strains (Cosgrove et al., 2003; de Kraker et al., 2011).

One of the alternative approaches to curing infections with antibiotic-resistant *S. aureus* strains is the use of lytic bacteriophages (Borysowski et al., 2011; Kaźmierczak et al., 2014). These are natural parasites of bacteria that can specifically infect and kill their host but are harmless to eukaryotic cells. Numerous cases of successful treatment of *S. aureus* infections with bacteriophages in animals (Iwano et al., 2018; reviewed by Barrera-Rivas et al., 2017) and in humans (d’Herelle, 1931a,b; MacNeal and Frisbee, 1936a,b; Sauve, 1936; Międzybrodzki et al., 2012; Fadlallah et al., 2015; Fish et al., 2016; reviewed by Kaźmierczak et al., 2014) have been described.

Several phages from the order *Caudovirales*, of a complex virion structure comprising head and tail, are obligatorily lytic on *S. aureus* hosts and cannot transfer bacterial DNA by transduction. Thus, they can potentially be used in therapy (reviewed by Łobocka et al., 2012). Most of them have a long, contractile tail and have been currently classified to the family *Herelleviridae* (Barylski et al., 2018). Some, less numerous and including only 14 isolates of sequenced genomes have a short tail (Vybiral et al., 2003; Kwan et al., 2005; Son et al., 2010; Kraushaar et al., 2013; Swift and Nelson, 2014; Wang et al., 2016; Gozdek et al., 2018). They belong to the *Picovirinae* subfamily of family *Podoviridae* (Lavigne et al., 2008). Those that infect *S. aureus* are much alike, despite being isolated in different geographical regions (Kraushaar et al., 2013), and have been affiliated to the *Rosenblumvirus* genus, subfamily *Picovirinae* (formerly 44AHJD-like phages or 68-like phages) (Lavigne et al., 2008; Gozdek et al., 2018).

The best known representative of *Picovirinae* is a model *Bacillus* phage phi29. However, the similarities between phi29 and the staphylococcal *Picovirinae* are limited to a similar genome size (17–20 kb) and tail morphology, partially conserved synteny between the genomes and the presence of terminal DNA repeats, protein-primed DNA replication and also structural homologies between certain proteins (Vybiral et al., 2003; Lavigne et al., 2008; Kraushaar et al., 2013). Functions of about half of the proteins encoded by the staphylococcal phages could be predicted based on their homology. The major head protein, receptor binding protein (RBP) and two virion proteins of unknown function were identified by proteomic studies (Uchiyama et al., 2017; Štveráková et al., 2018).

The interactions of the staphylococcal *Picovirinae* phages with their host cells are only beginning to be understood. Their adsorption to the cell surface requires a specific glycosylation pattern of the wall teichoic acid (WTA), which depends on the activity of *tarM-* or *tarS*-encoded glycosyltransferases catalyzing α-*O*-GlcNAcylation and β-*O*-GlcNAcylation of WTA, respectively (Li et al., 2015). Studies on the adsorption requirements of various *Rosenblumvirus* phages and the results of phylogenetic analysis of their RBPs allowed distinguishing two subgroups: phages that require β-*O-*GlcNAcylation of WTA for adsorption (SAP-2, SLPW, S13′, SCH1, Psa3, GRCS, 44AHJD, P68 and 66), and phages that adsorb to α-*O*-GlcNAcylated as well as β-*O*-GlcNAcylated WTA (S24-1 and BP39) (Li et al., 2015; Uchiyama et al., 2017). Consistent with the adsorption-promoting TarS-mediated WTA modification and adsorption-inhibiting TarM-mediated WTA modification some phages of subgroup I have also been shown to infect strains of *Staphylococcus xylosus* and *Staphylococcus equorum*, which encode homologs of *S. aureus* TarS but lack genes encoding homologs of TarM (Li et al., 2015). A phage tail lysin contributes to the penetration of phage DNA into the host cell. Another protein is associated with the DNA ends (Vybiral et al., 2003). Based on the homologies between DNA polymerases and between certain structural proteins of phi29 and *Rosenblumvirus* phages the lytic development of the latter is assumed to be similar to that of phi29, albeit it has not been studied. Cell lysis allowing phage release depends on phage endolysin, whose gene is located within a cluster of morphogenesis genes, unlike in phi29 (Lavigne et al., 2008).

Studies on the antistaphylococcal activity of *Rosenblumvirus* genus phages *in vivo* are sparce. One of these phages, S13’, was demonstrated to have a life-prolonging effect on *S. aureus*-infected silkworm larvae and protected about 40–50% of mice infected with *S. aureus* from death, including the death from lung-derived septicemia (Takemura-Uchiyama et al., 2013, 2014). Endolysin of another phage, SAP-2, was active in decreasing the biofilm mass of various *S. aureus* and *S. epidermidis* strains, as estimated in visual assay experiments (Son et al., 2010). Its lytic activity against various strains was wider than that of the parental phage. Little is known about the phage–bacteria interactions leading to severe limitation of bacterial growth without, however, threatening the phage with extinction due to elimination of its host. To fill in this gap we characterized here an additional phage of this genus, phiAGO1.3. We have identified its properties contributing to the wide host-strain range, and show its atypical strategy of co-existence with the host. Additionally, we demonstrate that this strategy not only does not preclude the antistaphylococcal activity of phiAGO1.3 *in vivo* but even leads to the selection of bacterial mutants of attenuated virulence.

## Materials and Methods

### Phage, Bacterial, and Nematode Strains and Culture Conditions

**FIGURE 1:**
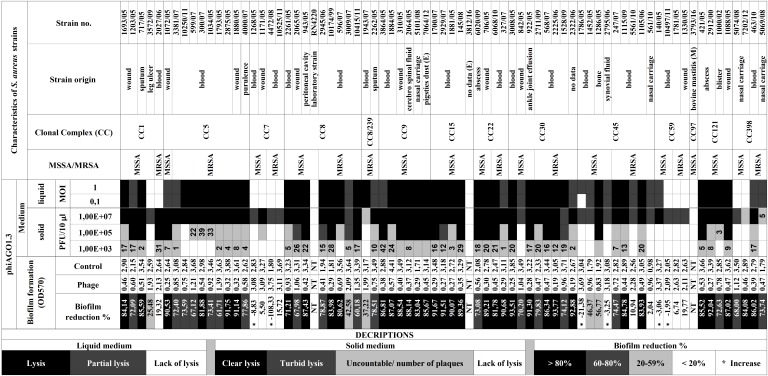
Activity of bacteriophage phiAGO1.3 in lysis of *Staphylococcus aureus* strains of various clonal complexes and in the destruction of their biofilms. Strain numbers are as in the MICROBANK collection, with the exception of laboratory strain RN4220. Environmental and bovine mastitis *S. aureus* isolates are marked by (E) and (M), respectively. Density changes of liquid cultures of *S. aureus* upon addition of phiAGO1.3 are exemplified in Supplementary Figure 3.

*Staphylococcus aureus* podovirus vB_SauP_phiAGO1.3 (phiAGO1.3) used in this study was isolated earlier by us from Warsaw sewage (Golec et al., 2011; Gozdek et al., 2018). However, using primers specific for temperate phages of various integrase types (Goerke et al., 2009) we identified a prophage/phage of Φ55 integrase type in the bacterial strain used for phiAGO1.3 propagation (2064/05), and in the phiAGO1.3-derived lysates of this strain (data not shown). To avoid contamination with this phage, strain 80wphwpl, which had been cured of active prophages and plasmids and whose genomic sequence had been determined (Łobocka et al., 2016) was used for phiAGO1.3 propagation in the present study. The monoclonality of the starting phiAGO1.3 preparations obtained from 80wphwpl cells was confirmed by negative results of PCR with a primer pair specific for the 2064/05-derived prophage (data not shown). Another phage used in this study, A5W, is a representative of *Kayvirus* genus and was described previously (Łobocka et al., 2012). The list of *Staphylococcus* spp. reference strains and *S. aureus* strains used in this study is given in Supplementary Table 1 and Figure 1, respectively. Of 75 *S. aureus* strains used, 74 strains came from the MICROBANK collection of the National Medicines Institute (NMI), Warsaw, Poland. Sixty six of them comprised clinical strains isolated from inpatients between 1999 and 2011. Eight of them were from nasal colonization in human (*n* = 5), from environmental samples (*n* = 2), and from bovine mastitis (*n* = 1). The isolates were characterized, at the phenotypic and molecular level; they represented 14 genetic lineages (MLST-Clonal Compexes [CCs]: CC1, CC5, CC7, CC8, CC8/239, CC9, CC15, CC22, CC30, CC45, CC59, CC97, CC121, and CC398). Thirty five isolates resistant to methicillin (MRSA) were assigned to 27 MRSA clones. They included hospital-, community- or livestock-associated representatives of 10 CCs (CC1, CC5, CC7, CC8, CC8/239, CC22, CC30, CC45, CC59, and CC398). Methicillin-susceptible *S. aureus* (MSSA) isolates (*n* = 39) belonged to all CCs with the exception of CC8/239. *S. aureus* strain RN4220 (CC8) is a restriction-modification deficient and prophage-free derivative of NCTC8325 described previously (Kreiswirth et al., 1983; Nair et al., 2011). Bacteria were grown at 37°C with constant shaking (200 rpm) in Luria-Bertani broth (LB; Difco), in brain heart infusion broth (BHI; Oxoid) or in trypticase soy broth (TSB; Difco), as indicated.

A standard laboratory *Caenorhabditis elegans* strain Bristol N2 (wild-type) was used as a model host for *S. aureus* infection in all experiments. It was obtained from the Caenorhabditis Genetics Center (CGC), University of Minnesota. The nematodes were maintained on NGM agar medium and fed *E. coli* strain OP50 as described elsewhere (Sulston and Hodgkin, 1988).

### Phage Propagation

To ensure the lack of contamination of phiAGO1.3-containing cell lysates with temperate phages or plasmid DNA the phage that was originally obtained by propagation in *S. aureus* 2064/05 (Gozdek et al., 2018) was passaged several times from a single plaque through *S. aureus* 80wphwpl, and lysates were verified for the absence of 2064/05-derived phage DNA by PCR with 2064/05-prophage (integrase type Φ55) specific primer pair (Goerke et al., 2009). Only confirmed monoclonal lysates were used for phiAGO1.3 propagation on a larger scale in 80wphwpl cells.

### Phage Purification

Cell lysate containing phages (50 ml, 10^11^ PFU/ml) was filtered through 0.22-μm pore size membrane (MILLEX^®^ GS) and treated with RNase A (PureLink RNase, Invitrogen) and DNase (Turbo DNase, Ambion) (final concentration 20 μg/ml of each) at 37°C for 2 h. Next, the lysate was supplemented with polyethylene glycol (PEG6000) and NaCl to final concentrations of 10% and 1 M, respectively, and incubated on ice for 24 h. Samples were centrifuged (10,000 × *g*, 30 min, 4°C) and the pellet was resuspended in phosphate buffered saline (PBS: 10 mM Na_2_HPO_4_, 137 mM NaCl, 2.7 mM KCl, 2 mM KH_2_PO_4_ at pH 7.4).

### Determination of Bacteriophage Host Range With a Spot Test

Overnight cultures of various *S. aureus* strains in LB medium (0.2 ml) were supplemented with CaCl_2_ and MgSO_4_ to the final concentration of 2.5 mM each, mixed with 1 ml LB and 8 ml molten LCA (55°C), overlaid on LB agar medium in Petri dishes, and left to solidify. Lysates containing phages were standardized by diluting in LB to obtain the titer of 10^8^, 10^6^, and 10^4^ PFU/ml. Ten microliters of each lysate at each dilution was spotted on the cell layer of each strain. Plates were incubated overnight at 37°C, and the number and morphology of plaques were determined.

### Assays of Bacteriophage Lytic Activity in Liquid Cultures of *S. aureus*

Overnight cultures of various *S. aureus* strains were diluted 1:100 in fresh LB medium supplemented with CaCl_2_ and MgSO_4_ (2.5 mM each). Aliquots of each culture (190 μl) were added to wells of sterilized honeycomb plates and incubated in a Bioscreen C Microbiology Plate Reader (Growth Curves USA, Piscataway Township, NJ, United States) at 37°C until the optical density (OD_600_) reached ca. 0.2. The cultures were then supplemented with the phage at MOI of about 0.1 and 1 by the addition of 10 μl of an appropriately diluted lysate or LB (control samples). The cultures were left for 10 min at room temperature (RT) without shaking to allow for phage adsorption, and then were incubated as above with medium intensity shaking for 24 h. The optical density of the cultures was measured during the whole experiment in 15-min intervals.

### Assay of Bacteriophage Influence on Preformed *S. aureus* Biofilms

The ability of phiAGO1.3 to disrupt preformed *S. aureus* biofilm was assayed as described previously (Stepanovic et al., 2000), with some modifications. *S. aureus* scraped from fresh LB agar plate was inoculated in 1 ml of BHI containing 2% sucrose, incubated overnight at 37°C without shaking and diluted 1:100 in fresh medium. Aliquots of bacterial suspension (100 μl) were transferred to wells of sterile 96-well flat-bottom polystyrene plates (Greiner Bio-One, Germany) containing a preformed fibrinogen layer (50 μg fibrinogen in 100 μl of water per well, incubated for 24 h at 4°C and carefully aspirated). Bacterial biofilm was allowed to form for 24 h at 37°C without agitation. The wells were then carefully washed twice with 200 μl of phosphate buffered saline (PBS). Next, 100 μl of diluted bacterial lysate containing phage (10^8^ PFU/ml) or LB (control) was added, the plates were incubated for 24 h as above and washed twice with PBS. The biofilm was fixed with 200 μl of 99% methanol for 15 min, drained, left to dry at 50°C for 1 h, and then stained with 0.01% crystal violet (CV; 200 μl) for 15 min. Excess CV was removed by washing three times with water and the wells were air-dried. The amount of CV bound by the biofilm was quantified by solubilizing in 200 μl of 33% acetic acid for 10 min followed by determination of the optical density of the obtained solution at 570 nm using a microplate reader (Bio-tek Synergy^TM^ HT).

### Killing Assay

The fraction of bacteria surviving phage infection was determined by killing assay, as described previously (Carlson, 2004; Abedon, 2017), with some modifications. Briefly, 80wphwpl culture (10 ml, OD_600_ of about 0.4) was infected with phiAGO1.3 at MOI 1 and 10 and left for 15 min at RT to allow for phage adsorption. The same volume of uninfected culture was used as a control. Samples were placed on ice, immediately serially diluted 10-fold in cold LB and spread on LB agar plates. The plates were incubated at 37°C for 24 h and bacterial colonies formed were counted. The fraction of surviving cells was calculated based on the ratio between the number of colonies formed by cells from infected and uninfected cultures.

### Identification of Phage-Releasing Colonies

Single *S. aureus* colonies recovered following phage infection described as above, were transferred onto a top agar overlay of strain 80wphwpl, incubated overnight at 37°C and checked for the presence of a lysis zone – a hallmark of spontaneous phage release. The presence of phiAGO1.3 was additionally verified by PCR with phage-specific primers. Briefly, candidate colonies were suspended in 100 μl of ice-cold water, and 1 μl of the suspension was used as a source of template for the DNA amplification with primers OMLO707 5′-TGCATGGCTTGTTTTGCTAAAGC-3′ and OMLO708 5′-ACAAGCWGGACAACCGTCTTGGT-3′. PCR conditions were as follows: 95°C for 15 min (95°C for 30 s, 55°C for 30 s, 75°C for 1 min) × 30, 75°C for 7 min.

### Determination of Phage Carrier State Stability

To evaluate the stability of the phage carrier state the fraction of cells retaining phiAGO1.3 following consecutive cell divisions was determined. Briefly, phage-releasing colony (designated as FTpt further in this manuscript) identified as above was suspended in 100 μl of cold water, placed on ice, immediately serially diluted 10-fold in cold LB and spread on LB agar plates. The plates were incubated at 37°C for 24 h, and colonies formed (designated as first generation: G_1_) were transferred onto a top agar overlay of strain 80wphwpl. After overnight incubation at 37°C, the percentage of phage-producing colonies (FTpt) in G_1_ population was determined. The procedure was repeated six times starting each time from cells of one phage-producing colony.

### Evaluation of phiAGO1.3 Efficacy in Curing *S. aureus-*Infected Nematodes

The ability of phiAGO1.3 to cure nematodes infected with *S. aureus* 80wphwpl was assayed as previously described (Łobocka et al., 2014b), with some modifications. An overnight tryptic soy broth (TSB) bacterial culture was diluted 1:100 in fresh medium and 100 μl was spread on a 5.5-cm diameter tryptic soy agar (TSA) plate and incubated at 37°C for 24 h. *C. elegans* larvae at T4 stage taken from a synchronized culture were transferred to a lawn of *S. aureus* cells and incubated at 25°C for 24 h. The infected nematodes were then rinsed from the plates, placed on a nylon mesh (CellMicroSieves, BioDesign) with 10-μm pores, washed twice with 5 ml of LB medium, and transferred onto a TSA plate. Then, they were covered with 5 ml of phage lysate (10^9^ PFU/ml) or LB medium (control), and incubated at RT for 1 h. The nematodes were then washed on a nylon mesh as above, transferred onto a TSA plate and left to dry. Twenty worms were picked randomly using a platinum wire pick and transferred to a fresh TSA plate. The plates were incubated at 25°C and scored for live and dead worms in 24-h intervals.

### Isolation of *S. aureus* DNA

Bacteria were harvested from overnight cultures (3 ml) by centrifugation (10,000 × *g* for 10 min) and resuspended in 200 μl of TE buffer (10 mM Tris-HCl, 1 mM EDTA, pH 8.0). Lysostaphine (Sigma) and RNase (Invitrogen) was added to 200 and 10 μg/ml, respectively, the sample was shaked vigorously and incubated at 37°C until the culture lysed. DNA was extracted using the EXTRACTME kit for genomic DNA isolation from bacteria (Blirt DNA-Gdańsk, Poland), according to manufacturer’s guidelines. DNA quality and quantity was checked by agarose gel electrophoresis.

### *S. aureus* Genomic DNA Sequencing, and Sequence Assembly and Analysis

*Staphylococcus aureus* genomic DNA was sheared mechanically to ∼600–700 bp and used for Paired-End TruSeq-like library construction using a KAPA Library preparation kit (KAPA/Roche, Basel, Switzerland) following manufacturer’s instructions. The library was sequenced in the paired-end mode (v3, 600 cycle chemistry kit) on a MiSeq instrument (Illumina, San Diego, CA, United States). Sequence reads were filtered by quality using the ultra-fast FASTQ preprocessing toolkit, fastp version 0.14.1 (Chen et al., 2018^1^). As an additional stage, performed only for the clone of control 80wphwpl strain used in this study (designated as 80wphwpl_v1), long reads were generated to aid genome assembly using a MinION nanopore sequencing instrument (Oxford Nanopore Technologies, Oxford, United Kingdom). Approximately 1 μg of unsheared DNA was used for library preparation using the ONT Rapid Barcoding sequencing kit (SQK-RBK004). Nanopore sequencing was performed using the NC_48h_Sequencing_Run_FLO-MIN106_SQK-RBK004 protocol (MinKnow version 2.2) and R9.4.1 MinION flowcell. Raw nanopore data was basecalled using Albacore v2.3.1 (Oxford Nanopore Technologies, Oxford, United Kingdom). Following quality filtering and sequencing adapter removal using Porechop^2^ 17,527 barcoded reads remained. The median read length of the obtained dataset (132,564,821 of bases in total) was 5,649 bases. Long nanopore reads were assembled in a hybrid mode with the Illumina data using Unicycler v.0.4.6 (Wick et al., 2017). Genome hybrid assembly resulted in a single circular replicon. The suspected errors in the genome assembly, especially in genes containing tandem repeats, were verified by PCR amplification of relevant DNA fragments followed by Sanger sequencing on an ABI3730xl Genetic Analyzer (Life Technologies, United States) using BigDye Terminator Mix v. 3.1 chemistry (Life Technologies, United States). Missassemblies were further corrected using Seqman software (DNAStar, United States) to obtain the complete nucleotide sequence of 80wphwpl_v1 (2,732,618 bp). This newly generated consensus sequence of the parent strain genome was used as a template for the genomic sequence assembly of the *S. aureus* isolates resistant to infection with phiAGO1.3. Desktop software Geneious 11.0.4 (Biomatters, Ltd., Auckland, New Zealand) was used for the assembly. The alignments obtained served for the detection of differences between the mutants and the parental strain. Additional inspection of the sequence reads in the alignments served to exclude the ambiguities in the consensus sequence of the reference and mutant strains in the regions of detected mutations.

## Results

### Basic Characteristics of phiAGO1.3

Bacteriophage phAGO1.3 was originally isolated from sewage as an obligatorily lytic phage capable of infecting most *S. aureus* strains from a small collection of clinical isolates (Gozdek et al., 2018).

Measurement of typical phage growth parameters revealed that phiAGO1.3 could be a potential candidate for therapeutic applications. It can adsorb quickly to its host cells and has a short latent period (∼30 min) (Supplementary Figure 1). Its burst size was estimated to be about 35. Additionally, its titer in the lysate did not change significantly after 1 h of incubation at 37 or 50°C or at pH from 5 to 9 (Supplementary Figure 2). Only incubation at temperatures of 60°C or higher or at pH below four or above 10 caused a drastic decrease of the phage activity. The optimal MOI for phiAGO1.3 propagation in *S. aureus* 80wphwpl was determined to be 0.01 (data not shown).

### Coding Potential of phiAGO1.3 and Analysis of Its Structural Proteome

The genome of phiAGO1.3 encodes 20 proteins (Gozdek et al., 2018). Their homologs are found in various *Picovirinae* that infect *S. aureus.* Phages GRCS and PSa3, which, respectively, encode eight and seven of them, appear to be the closest relatives of phiAGO1.3 at the protein sequence level (Supplementary Table 2). Close homologs of all phiAGO1.3 proteins are also encoded by prototypical *Rosenblumvirus* phages 44AHJD or 68. Eleven of the phiAGO1.3 proteins are virion components, as identified by a mass spectrometry analysis of tryptic peptides of whole virions (Supplementary Table 3). Homologs of nine of them, including the major capsid protein, two tail proteins, lower and upper collar protein, RBP and tail amidase, have been identified or predicted previously as virion proteins of related phages (Vybiral et al., 2003; Takemura-Uchiyama et al., 2013; Uchiyama et al., 2014; Štveráková et al., 2018). The two additional virion proteins of phiAGO1.3 identified in this work (ORF01 and ORF07) are present in virions in a small amount as suggested by the small number of their peptides in analyzed samples (Supplementary Table 3). They have counterparts in related staphylococcal phages, but do not have apparent homologies to proteins of known function. One of them could act as the terminal protein priming the phage DNA replication, by analogy to phi29. DNA termini of phage 68, closely related to phiAGO1.3, have been shown to be covalently bound to a protein (Vybiral et al., 2003). A virion protein of phiAGO1.3 that might somehow contribute to the host specificity is ORF20. It appears to be, besides the tail fiber protein (ORF13), the most variable part of *S. aureus Rosenblumvirus* phages’ virions. It is a highly acidic protein, which in phiAGO1.3 contains six repeats of ES/LTE amino acid sequence motif. The variability of ORF20 and its homologs in *S. aureus* phages results partly from the differences in the number of these repeats.

### Determination of phiAGO1.3 Lytic Spectrum

Certain polyvalent staphylococcal phages infect representatives of various *Staphylococcus* sp. Thus, we tested the sensitivity of 12 selected type strains of *Staphylococcus* spp. other than *S. aureus* to phiAGO1.3 (Supplementary Table 1). The phage infected productively one of the two tested strains of *Staphylococcus hyicus*, which like *S. aureus* belongs to coagulase-positive staphylococci, and, surprisingly, a type strain of a coagulase-negative *Staphylococcus*–*Staphylococcus lugdunensis* (DSM No. 4804, data not shown).

To evaluate the potential of phiAGO1.3 to control *S. aureus* we tested its lytic activity against agar-embedded cells, planctonic cells and biofilms of 75 *S. aureus* clinical isolates from a diversified collection (Figure 1). The phage infected productively 55 (73%) of those strains when tested by a spot test, as indicated by the formation of a lysis zone at high phage doses (10^7^–10^5^ PFU/10 μl), and single plaques or confluent plaques with some borders between them visible at lower phage doses. In the assays of the phage lytic activity in liquid culture a significant decrease of the culture density was observed for 61 (81%) of strains upon addition of phiAGO1.3 (Figure 1 and Supplementary Figure 3). This difference in the number of susceptible strains is likely caused by the low efficiency of infection of some of them (e.g., 10250/11) resulting from the action of their restriction systems. Conceivably, the initial infection required some population phages to break the restriction barrier of the infected strain. This in turn could lead to the lytic development of the phage and the release of appropriately modified progeny phages able to infect cells of this strain efficiently. Such effect of a lack of strain-specific DNA modifications in the phage DNA has been observed previously for other staphylococcal obligatorily lytic phages (see, e.g., Pantůček et al., 1998).

The pattern of the culture density changes upon addition of phiAGO1.3 differed for different *S. aureus* strains. In the case of 44 strains all or nearly all cells in the culture underwent lysis (Supplementary Figure 3A). For the remaining 17 strains the drop in culture density, even at MOI 1, was small and followed by a plateau (Supplementary Figure 3B) suggesting that productive infection of some cells may be accompanied by the continuous growth of uninfected or resistant cells.

Fourteen of 75 strains tested did not lyse in liquid cultures upon addition of phiAGO1.3 (Figure 1 and Supplementary Figure 3C). Additionally, we could not obtain single plaques in the spot test with those strains at any phage dose, despite the presence of lysis zones at high phage doses. Notably, for the latter strains similar lysis zones were obtained using suspensions of purified phage, indicating that they are not caused by free lysins or bacteriocins but by the phage itself. The observed effect could be caused by so called “lysis from without” or by the activity of bacterial phage defense systems causing death of infected cells to stop the phage spread. Apparently, phiAGO1.3 can adsorb to cells of those strains and penetrate their cell envelopes, but the infection is not productive.

Only three *S. aureus* strains of the entire set tested (RN4220, 3812/16, and 3793/16) appeared to be insensitive to phiAGO1.3 as indicated by the lack of any lysis in the spot tests and in liquid culture treated with the phage. Conceivably, their resistance is due to a lack of appropriate phage receptors.

In the majority of cases *S. aureus* forms biofilms at the site of infection (Moormeier and Bayles, 2017). Thus, we determined the effect of phiAGO1.3 on the biofilm mass of the strains tested (Table 1 and Supplementary Figure 4). In the case of the strains which could not be infected productively in the former assays, phage addition to the preformed biofilm did not influence the biofilm biomass or caused an increase (from 21% to as much as 108%) or a slight decrease of the biofilm biomass. For all the strains that were productively infected with phiAGO1.3, a significant decrease of biofilm biomass (mostly by over 80%) was observed (see Figure 1 and Supplementary Figure 4).

**Table 1:** Number of target sites for the *Staphylococcus aureus* R-M systems of Type I and Type II of known specificities in phiAGO1.3 genome.

Clonal complex	HsdS subunit name in REBASE	Generic name	Recognition sequence	Number of targets in phiAGO1.3 DNA	I/T^∗^
CC1	S. SauMW2I S. SauMW2II	CC1-1 CC1-2 (CC8-2)	CCAY-5-TTAA CCAY-6-TGT	1 0	4/5
CC5	S.SauN315II S.SauN315I	CC5-2 CC5-1 (CC8-2)	CCAY-6-GTA AGG-5-GAT	3 0	10/10
CC8#				0	7/8
CC15	S.SauL3150RFAP	CC15-1	CAAC-5-RTGA	0	4/5
CC22	S.Sau5096I	CC22-1	AGG-6-TGAR	5	4/4
CC30	S.SauMRSII S.SauMRSI	CC30-1 CC30-2	GWAG-5-GAT GGA-7-TCG	0 0	8/8
CC45	S.Sau347I	CC45-1	GWAG-6-TAAA	0	9/9
CC51	S.SauL30RFAP	CC51-1	GGA-6-CCT	0	NT
CC59	S.SauSA40ORF370P	CC59-1	GGA-6-RTGT	1	1/4
CC72	S.SauCN10RF415P S.SauCN10RF1757P	CC72-1 CC72-2	GARA-6-RTGT GGA-7-TGC	1 0	NT
CC75	S.Saull32ORF3780P S.Saull32ORF16570P	CC75-1 CC75-2	CAAG-5-RTC CNGA-7-TTYG	2 0	NT
CC93	S.SauJKDIII S.SauJKDII S.SauJKDI	CC93-3 CC93-2 CC93-1	GAAG-5-TAC or complement GGHA-7-TCG CAG-6-TTC	0 3 1 3	NT
CC97	S.SauC01791ORFAP	CC97-1	CCAY-6-RTC	0	0/1
CC133	S.Saul330RF451P S.Saul330RF1794P	CC133-1 CC133-2	CAG-5-RTGA GGA-7-TTRG	1 0	NT
CC398	S.SauSTORF499PCC398-l CC398-1		ACC-5-RTGA	5	3/4
CC873	S.Sau323260RFAP	CC873-1	GAG-6-GAT	5	NT
Sau3A Sau96I	R-M Type II enzymes		GATC GGNCC	2 0	

*^∗^I/T indicates the number of infected strains as compared to the number of tested strains. ^#^ indicates that the sequences are the same as CC8-2.*

### Properties of phiAGO1.3 Contributing to Its Wide *S. aureus* Strain Range

*Staphylococcus aureus* protection from invading foreign DNA and from horizontal gene transfer between different lineages is provided mainly by Type I DNA restriction-modification (R-M) systems, which were generally designated as *Sau1* although their methylation specificity varies between different CCs (Waldron and Lindsay, 2006). The ability of phiAGO1.3 to infect productively strains of at least 12 CCs implies that either it lacks the relevant target sequences in its DNA or encodes a potent antirestriction system. The target sequences of certain *S. aureus* R-M systems have been identified (McCarthy and Lindsay, 2010, 2012; McCarthy et al., 2012; Roberts et al., 2013; Monk et al., 2015; Chen et al., 2016; Cooper et al., 2017). The phiAGO1.3 genome carries no recognition sequences for CC1-2, CC8, CC30, CC45, CC51, CC72-2, CC75-2, CC93-3, CC97 and CC133-2 *Sau1* R-M systems, and only single recognition sequences for CC1-1, CC59, CC72-1, CC93-2, and CC133-1 *Sau1* R-M systems (Table 1). The DNA cleavage by restriction endonucleases of classical Type I R-M systems requires a collision of two enzymes translocating toward each other (Studier and Bandyopadhyay, 1988; Dreier et al., 1996; Jindrova et al., 2005; Šišáková et al., 2013). Thus, a single site does not suffice for the digestion of infecting linear phage DNA. In addition to Type I R-M systems several *S. aureus* strains encode Type II R-M systems such as Sau3A, Sau96I, and isoschizomers of SmlI that recognize 5′-GATC, 5′-GGNCC, and 5′-CTYRAG sequences, respectively, and cleave them if they are not methylated (Sussenbach et al., 1976, 1978; Stobberingh et al., 1977; Szilák et al., 1990; Godány et al., 2004). The genome of phiAGO1.3 contains only two GATC sequences and no GGNCC sequences. Taken together these results indicate that the elimination of certain restriction enzyme recognition sites is the dominant phiAGO1.3 strategy to avoid the activity of host R-M systems.

### Killing Efficacy of Its Bacterial Host by phiAGO1.3 and the Establishment of Phage Carrier State

The efficacy of a given phage to kill the infected bacteria, so called killing efficacy, is a critical parameter in the evaluation of phage therapeutic potential (Carlson, 2004; Łobocka et al., 2014a; Abedon, 2017). The killing efficacy reflects a sum of the bacteria killed as a result of productive phage propagation and those killed without the productive propagation, e.g., by the induction of bacterial apoptosis. Infection of *S. aureus* 80wphwpl with phiAGO1.3 at MOI 1 caused killing of about 74% of cells, whereas the infection at MOI 10 caused killing of 93% of cells.

**FIGURE 2:**
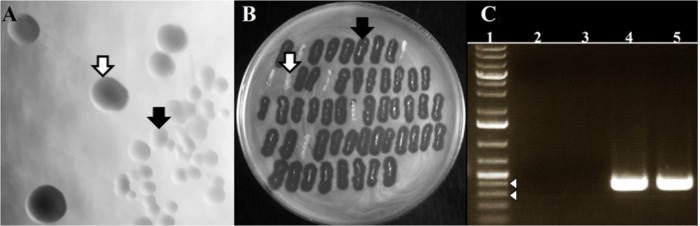
Differentiation of colonies formed by phiAGO1.3-infected cells of *S. aureus* 80wphwpl. **(A)** Different colony morphotypes recovered following phiAGO1.3 infection: wild-type colonies, designated as WTpt (white arrow) and small, transparent colonies designated as FTpt (black arrow). **(B)** Identification of phage-releasing colonies. Single *S. aureus* colonies recovered following phage infection were picked onto a layer of 80wphwpl cells in top soft LB agar, incubated overnight at 37°C, and checked for the presence of a lysis zone (WTpt, white arrow; FTpt, black arrow). **(C)** Presence of phiAGO1.3 in colonies surviving phage infection. PCR with phage-specific primers was used to identify phage DNA. Lanes represent: (1) 1-Kb Plus DNA Ladder (Invitrogen), (2) PCR with no template, (3 and 4) Colony PCR with WTpt and FTpt colonies as a source of template, respectively, (5) PCR with phiAGO1.3 as template. White arrowheads indicate 400- and 300-bp marker.

Irrespective of the MOI used for the infection, the bacteria that survived the infection formed colonies of two morphological types: those resembling colonies of non-infected cells (designated here as WTpt, wild-type colonies after phage treatment) and small, transparent colonies (designated as FTpt, frail type colonies after phage treatment) (Figure 2A). The fraction of the latter varied from 20 to 40%. While the transfer of cells from the WTpt colonies to a layer of 80wphwpl cells embedded in soft agar did not cause any effect, the transfer of cells from the FTpt colonies caused the formation of lysis zones of indicator bacteria (Figure 2B). The lysis was observed even after the FTpt cells were washed several times before plating. Colony PCR of FTpt bacteria with primers specific for phiAGO1.3 revealed the expected 353-bp product, indicative of the presence of phiAGO1.3 (Figure 2C). No product was obtained with WTpt colonies. Clearly, the phenotype of FTpt colonies is associated with the presence of phiAGO1.3 phage or phiAGO1.3 DNA.

### The Establishment of Equilibrium Between Host Bacteria and phiAGO1.3 in Liquid Cultures

**FIGURE 3:**
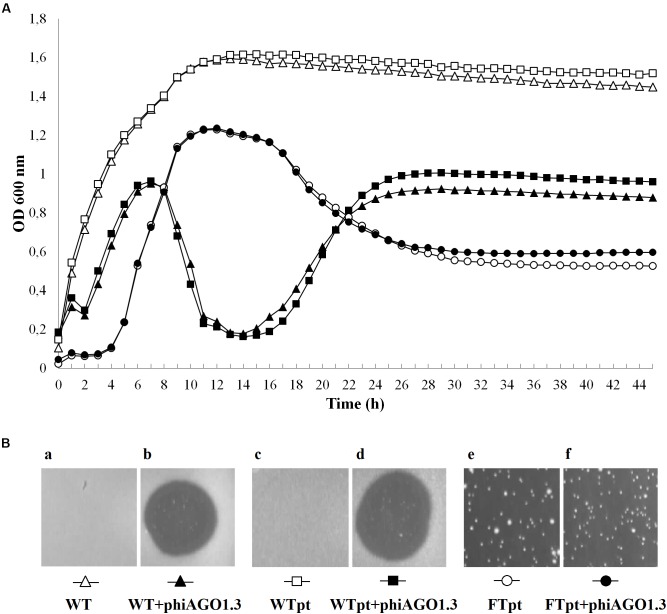
Properties of *S. aureus* 80wphwpl (WT) and its derivatives recovered from phiAGO1.3-treated cultures (WTpt and FTpt). **(A)** Optical density changes of liquid cell cultures of WT (empty triangles), WTpt (empty squares) and FTpt (empty circles) colonies grown without phiAGO1.3 added and upon addition of phiAGO1.3 (filled triangles, squares, and circles, respectively). Portions of LB medium (190 μl) in duplicate were inoculated with appropriate colonies to OD_600_ of about 0.1–0.2. One culture of each pair was supplemented with 10 μl of phiAGO1.3 suspension in LB (10^9^ PFU/ml) and one (control) with 10 μl LB. Cultures were grown in Bioscreen C at 37°C with shaking. **(Ba-f)** Sensitivity of WT and WTpt- and FTpt-derived *S. aureus* to phiAGO1.3, as measured by a spot test. FTpt-derived cells lysed spontaneously in the top agar (Be) and thus the site of phiAGO1.3 application in Bf is not visible.

The optical density of 80wphwpl cultures infected with phiAGO1.3 (10^7^ PFU/ml) began to decrease after 7 h and reached the lowest value (OD_600_ of about 0.2) at 15 h, and then increased to reach a plateau (OD_600_ of about 0.9) at about 25 h (Figure 3). The pattern of density changes in the culture of cells derived from WTpt colonies and infected with phiAGO1.3 was similar, consistent with the sensitivity of these cells to phiAGO1.3 (Figure 3Bd). In contrast, cultures derived from FTpt colonies behaved differently. In the absence of externally added phiAGO1.3 they grew poorly for the first few hours, then increased the optical density for the next 6 h up to OD_600_ ca. 1.2 and then decreased the optical density to reach a plateau (OD_600_ of about 0.6) at about 26 h (Figure 3A, empty circles). A similar pattern of density changes was observed in the FTpt-derived cultures when phiAGO1.3 (10^7^ PFU/ml) was added to the cultures at the beginning of the experiment. This lack of an effect of external phage suggested that either the bacteria in the culture were not accessible to the newly added phages or that the infection by the newly added phages was prevented by other means (Figure 3A, black circles). Cells of FTpt-derived cultures taken at the 19-th hour, when a significant decrease of culture density was observed (OD_600_ of about 0.9), formed colonies of WTpt and FTpt types, at ratios varying between apparently identical experiment repetitions (1:1, 2:1, 1:2) despite similar curves of culture optical density changes. The total number of cells in the culture at that time, as calculated from the sum of WTpt and FTpt colonies formed, was 10^8^ CFU/ml, while the total number of phages, as calculated by titration, was 10^9^ PFU/ml. In a sample taken at the 32-nd hour the total number of cells was 10^7^ CFU/ml and of phages – 10^9^ PFU/ml. Apparently, the phage and the bacteria can reach a kind of equilibrium in which they can stably coexist with each other.

We reasoned that the FTpt colonies could not increase in size and could not give rise to a growing liquid culture if they did not contain at least a fraction of cells able to grow and divide. Thus, we tested what kind of cells contribute to the formation of FTpt colonies. Single FTpt colonies (designated as G0 generation) were used as the starting material in these experiments. To decrease the probability of phage release and infection of new cells during cell handling and to slow down cell division, suspensions of cells of single FTpt colonies and dilutions were prepared in ice-cold water and the cells were immediately spread on LB plates (the whole procedure did not exceed 3 min). The FTpt progeny cells formed colonies of both the FTpt and WTpt types. To test whether and to what extent the segregation phenotype is inheritable the whole procedure of cell washing, dilutions and plating was repeated with each next passage of cells of FTpt colonies up to seven rounds. Regardless of the passage the fraction of cells forming FTpt colonies varied between 40 and 90% and did not show any tendency to decrease with subsequent passages. Colonies from all the passages studied were tested for the ability to release phiAGO1.3 by their application on a layer of 80wphwpl cells in soft LCA. All FTpt colonies released the phage, while no WTpt colonies did. Taken together our results demonstrate that FTpt colonies are a mixture of two types of bacterial cells and that the phage carrier state is inheritable. The recurrent segregation of FTpt colonies into phage-releasing cells and phage-free cells sensitive to phage infection ensures a stable co-existence of bacteriophage and its host bacteria.

### Isolation of Cells Resistant to Infection With phiAGO1.3

**FIGURE 4:**
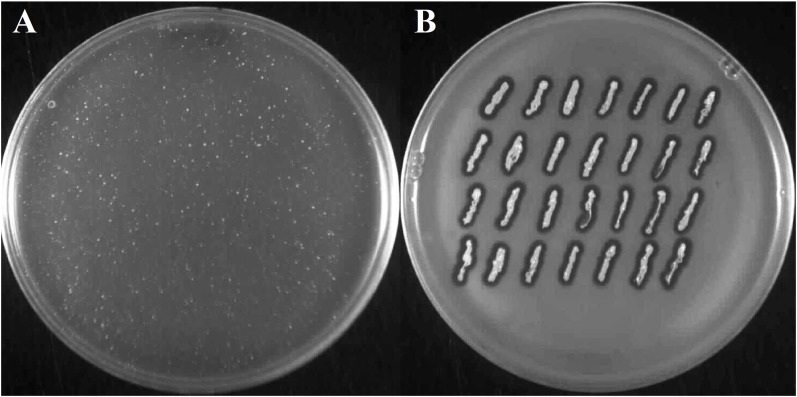
Emergence of phiAGO1.3-resistant clones among bacteria derived from FTpt colonies. **(A)** Growth of single FTpt-derived colonies on a layer of lysed FTpt-derived cells in soft agar. Single FTpt colony was suspended in LCA and spread on LB agar. Plates were incubated for 24 h at 37°C. Colonies formed were designated RevFT. **(B)** Identification of phiAGO1.3-releasing isolates among RevFT-derived bacteria.

When cells of FTpt colonies were suspended in molten LCA medium and poured onto solid LB medium, as is commonly done in the double-layer-agar assay of bacteriophage activity, they formed a lysis zone. However, throughout the lysis zone one could observe single bacterial colonies of various sizes (Figure 4A), similarly as in the case of the lysis zones obtained during phiAGO1.3 titration on a layer of 80wphwpl cells with the use of high-titer lysates.

To test whether these colonies release the phage like the FTpt colonies did, cells from the top of the colonies were transferred on a layer of 80wphwpl cells embedded in soft agar. Similarly, like as for the FTpt colonies, lysis zones of the indicator cells were formed around the cells of tested colonies. However, opposite to the test with the FTpt colonies, the intensive growth of bacteria was observed in the center of the lysis zones (compare Figures 2B and 4B). To distinguish these cells from cells of the parental FTpt colonies they were designated RevFT (reverse colonies of FTpt).

**FIGURE 5:**
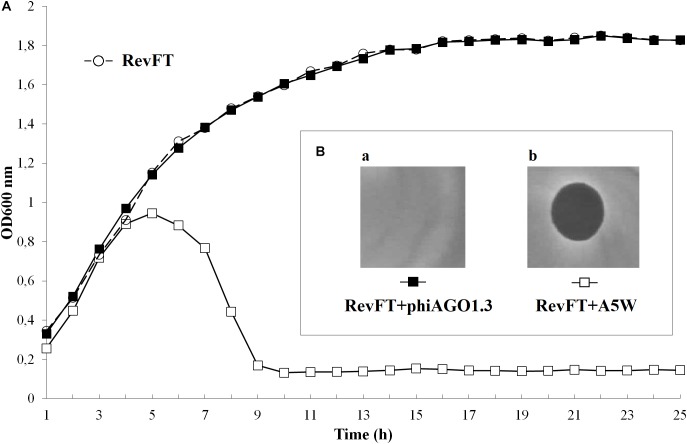
**(A)** Sensitivity of RevFT-derived bacteria to two unrelated phages, phiAGO1.3 and A5W of *Kayvirus* genus. Optical density changes of RevFT culture cells (white circles) upon addition of phage phiAGO1.3 (black squares) or phage A5W (white squares). **(Ba,b)** Sensitivity of RevFT cells to phiAGO1.3 and A5W, as measured by a spot test.

All or some WTpt or RevFT cells could potentially represent phage resistant clones. To verify this possibility they were tested for the sensitivity to infection with phiAGO1.3. Cells from WTpt turned out to be sensitive to phiAGO1.3, as indicated by the ability of phiAGO1.3 to form lysis zones on a layer of these cells in LCA (compare Figures 3Bd and 3Bb) and by the optical density drop of their cell cultures upon addition of phiAGO1.3 (Figure 3A). Most likely, WTpt represent the progeny of cells that avoided the encounter with phiAGO1.3, as happens in killing assay experiments (Łobocka et al., 2014a). The results were different for the RevFT colony-derived cells, as they did not lyse upon phiAGO1.3 application (Figure 5Ba). Clearly, RevFT are cells that have acquired resistance to phiAGO1.3 (Figure 5).

To check whether the resistance phenotype is stable, RevFT cells were purified by passaging through fresh medium, re-isolated from single colonies and tested for sensitivity to phiAGO1.3 by spotting diluted cell suspension on a layer of LCA soft agar containing phiAGO1.3 (10^9^ PFU/ml). The RevFT-derived cells formed colonies and their number was comparable to that formed by a phiAGO1.3 resistant strain, RN4220 (data not shown). Clearly, the resistance of RevFT cells to phiAGO1.3 is maintained even when their contact with the phage is interrupted.

Certain phages of the genus *Rosenblumvirus* require WTA for adsorption, similarly to the *Kayvirus* genus phages. However, the RevFT cells, despite being resistant to phiAGO1.3 remained sensitive to infection with the *Kayvirus* phage A5W, as indicated by the assays in solid as well as in liquid medium (Figures 5A [black squares] and 5Bb). Clearly, their resistance is not associated with a change of any surface or internal cell structure of *S. aureus* that could be essential for the sensitivity to A5W.

### Identification of RevFT Mutation Associated With the Resistance to phiAGO1.3

The stable inheritance of phiAGO1.3 resistance by the progeny of cells forming FTpt colonies prompted us to determine the genomic sequence of several RevFT clones and compare it with the sequence of the parental 80wphwpl strain (80wphwpl_v1 control clone). All nine RevFT isolates sequenced had the substitution of G by T at position 1252 of the *arl* gene. This substitution is a nonsense mutation changing a glycine codon (GGA) to a STOP codon (TGA), which results in the production of the ArlS protein depleted of 34 C-terminal amino acid residues. ArlS is a sensory histidine kinase of the two component *S. aureus* regulatory system ArlRS (Fournier and Hooper, 2000^3^). The premature termination of translation in the mutant occurs exactly prior to the conserved GLGL motif which is an essential part of the dimerization and phospho-acceptor domain of ArlS protein (pfam HATPase_c domain) – one of the essential units of the two-component transduction system (Fournier and Hooper, 2000; West and Stock, 2001). Consistently, the ArlRS system cannot be functional in the mutant.

### Therapeutic Potential of phiAGO1.3 to Cure Nematode Infection With *S. aureus* 80wphwpl

**FIGURE 6:**
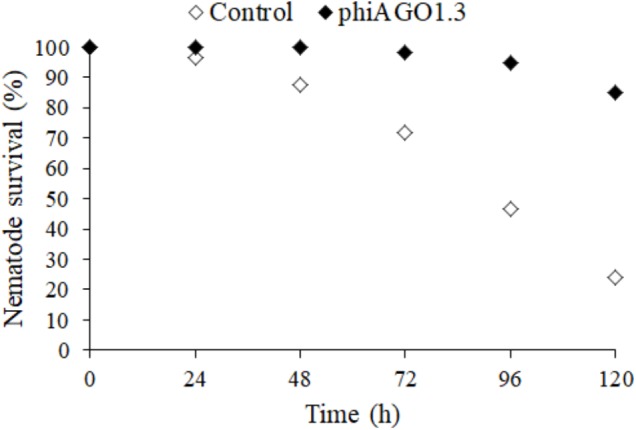
Antistaphylococcal activity of phiAGO1.3 *in vivo*. Percentage of surviving worms fed *S. aureus* 80wphwpl (untreated control, white diamonds) and treated with phiAGO1.3 (black diamonds). Each value is the mean of six samples from three experiments where the total number of worms was 120. The differences between experiments did not exceed 4% for untreated worms and 1% for phiAGO1.3-treated ones.

If an equilibrium between a phage and its host bacteria similar to that observed in liquid cultures could be also established in *S. aureus*-infected humans or animals during phage therapy it might preclude the use of phiAGO1.3 for therapeutic purposes. Thus, we tested whether phiAGO1.3 can rescue from death *C. elegans* infected with *S. aureus* 80wphwpl. Figure 6 show that 80wphwpl is pathogenic to *C. elegans*, causing gradual death of more and more nematodes each day so that only about 20% of them survived at 120 h post-infection. Additionally, none of them produced progeny. Opposite to that over 80% of the nematodes survived when they were treated with just one dose of phiAGO1.3 (10^9^ PFU/ml) 24 h after the infection. Moreover, ca. 100 h after the treatment with phiAGO1.3 they started to produce progeny, indicating the recovery of reproductive potential and thereby prompting us to stop the experiment. Taken together our results demonstrate that the ability of phiAGO1.3 to establish a stable mixed population with its bacterial host and to exist in equilibrium with the host under *in vitro* conditions does not negatively influence the therapeutic potential of phiAGO1.3.

### Nucleotide Sequence Accession Number

The genomic sequence of the *S. aureus* 80wphwpl WT, control clone (80wphwpl_v1) has been deposited in the GenBank under the Accession No. CP034098.

## Discussion

Carefully selected obligatorily lytic bacteriophages that efficiently kill bacterial pathogens may become the option of choice in the treatment of infections with antibiotic-resistant bacterial strains. However, safe introduction of a phage to therapy requires its biology and outcomes of interactions with its host bacteria *in vitro* and *in vivo* to be examined to the widest extent possible.

Here, we show that the polyvalent staphylococcal lytic podovirus phiAGO1.3 has properties that make it a potential candidate for therapeutic applications, identify factors that contribute to its wide strain range, and prove its therapeutic efficacy in curing a staphylococcal infection of a model nematode, *Caenorhabditis elegans*. Additionally, we demonstrate for the first time for any staphylococcal lytic phage, that phiAGO1.3 and its host bacteria can co-exist in a stable equilibrium which can be maintained owing to the presence in such mixed population of phage-carrier state cells. The ability to form phage-carrier state cells with its host not only does not cause the lack of efficacy of phiAGO1.3 as an antistaphylococcal agent *in vivo*, but also leads to the emergence of phage-resistant host mutants impaired in virulence.

The phiAGO1.3 virion morphology, genomic features and proteome place this phage among typical *Rosenblumvirus* phages, which infect *S. aureus* (Supplementary Table 2; Vybiral et al., 2003; Kwan et al., 2005; Son et al., 2010; Kraushaar et al., 2013; Takemura-Uchiyama et al., 2013; Swift and Nelson, 2014; Wang et al., 2016; Gozdek et al., 2018). The host range of only some of them has been studied and varied between 50 and 90% of the *S. aureus* strains tested (Son et al., 2010; Kraushaar et al., 2013; Takemura-Uchiyama et al., 2013; Wang et al., 2016). However, none of these host-range tests were conducted using the phage propagated in a prophage-free host. Thus, one cannot exclude that the plaques observed with certain strains could represent plaques of a prophage-derived temperate phage. To avoid this ambiguity, we determined the host range of phiAGO1.3 with various representatives of the genus *Staphylococcus* and representatives of 14 *S. aureus* clonal complexes using a prophage-free host for phage propagation. Of 75 *S. aureus* strains tested only some human isolates of CC1, CC59 and CC398, a bovine mastitis isolate of CC97 and isolates of CC7 were resistant to phiAGO1.3. However, despite the lack of productive infection of the majority of these resistant strains, phiAGO1.3 could cause their lysis from without, indicating that the resistance in not caused by a lack of appropriate phage receptors.

The ability of phiAGO1.3 to infect representatives of most of the *S. aureus* CCs requires bypassing of staphylococcal Type I R-M systems (*Sau1*) of different specificities. We show here that the main strategy of phiAGO1.3 to overcome the action of R-M systems of Type I and also Type II is the avoidance or sparcity of DNA sequences recognized by these systems in its genome. This strategy appears to be common to all *Rosenblumvirus* phages. They all lack in their genomes the GGNCC sequences (targets of Sau96I) and usually lack or only have single copies of sequences recognized by certain CC-specific variants of *Sau1* (data not shown).

The absence of the recognition sequences for the *Sau1* R-M systems of certain CCs could in principle be used as a predictor of the strain-range of a phage. Our results indicate that it is often, but not always, true. Some CC22 and CC15 strains tested were infected productively by phiAGO1.3 despite the presence of five recognition sequences for each of the respective *Sau1* systems in the phiAGO1.3 genome. Some phages encode antirestriction proteins protecting phage DNA from the host Type I R-M systems (Labrie et al., 2010; Samson et al., 2013; Iyer et al., 2017). Although phiAGO1.3 does not encode homologs of known antirestriction proteins, a role of one of its proteins of unassigned function in antirestriction cannot be excluded (Supplementary Table 3, Vybiral et al., 2003).

Some of the CC1 and CC59 strains tested were susceptible to lysis from without but still could not be infected productively by phiAGO1.3, despite a lack or the presence of only a single copy of recognition sequences for the respective *Sau1* systems in the phage DNA (Table 1). A likely reason for this could be the action of other R-M systems in these strains. Several *S. aureus* strains encode two related Type I R-M systems (Waldron and Lindsay, 2006; McCarthy and Lindsay, 2010; Monk et al., 2012; Lindsay, 2014) or contain additional R-M system genes on mobile elements, such as prophages, pathogenicity islands, or *SCC-mec* cassettes (Ito et al., 2004; Dempsey et al., 2005; Noto et al., 2008). Additionally, one cannot exclude a protective activity of CRISPR-Cas systems or abortive infection systems (Abi) (Chopin et al., 2005; Labrie et al., 2010), which also have been found in some strains of *S. aureus* (Yang et al., 2015; Cao et al., 2016; Depardieu et al., 2016). The staphylococcal Abi system involves the serine/threonine kinase Stk2 which is activated by phage proteins during lytic development and causes cell death by phosphorylating essential proteins. Stk2 protects the bacteria from the infection with all siphoviruses tested. Its influence on the sensitivity to podoviruses has not been studied.

Productive infection as well as lysis from without can serve as an indicator of successful phage adsorption to a bacterial cell. Accordingly, phiAGO1.3 could adsorb to all but three *S. aureus* strains tested. One of them is RN4220 whose WTA is modified by the TarM-mediated α-*O*-GlcNAcylation (Li et al., 2015). The inability of phiAGO1.3 to adsorb to RN4220 cells and the high homology of phiAGO1.3 RBP to the RBPs of subgroup I *Rosenblumvirus* phages (Supplementary Table 2) implies the requirement of WTA β-*O*-GlcNAcylation for phiAGO1.3 adsorption. What causes the sensitivity of certain *S. hyicus* and *S. lugdunensis* strains to phiAGO1.3 is unclear. We detected proteins of only partial homology to the *S. aureus* TarS (which mediates WTA β-*O*-GlcNAcylation) among predicted gene products of both infected strains (data not shown).

The β-*O*-GlcNAcylation of WTA is essential for the expression of methicillin-resistance in *S. aureus* and requires the function of TarS protein (Brown et al., 2012). This implies that MRSA strains are likely to adsorb *Rosenbslumvirus* genus phages of subgroup I. In support of that, all three strains that did not adsorb phiAGO1.3 in our tests are MSSA (Figure 1).

We show here that under certain conditions the exposure of *S. aureus* cells to phiAGO1.3 can lead to the establishment of a mixed population in which bacteria and bacteriophages are in an equilibrium, and remain in a stable ratio even following several serial passages – an equivalent of multiple generations. This interaction is reminiscent of so called phage carrier state cultures (PCSCs) (Lwoff, 1953). In the PCSC, phage-resistant and phage-sensitive cells co-exist in an equilibrium supporting a continuous growth of the bacterial as well as the phage population (Lwoff, 1953; Abedon, 2009). The bacteriophage carrier state has been described for over a dozen of bacteria (Hooton et al., 2016, and references therein), but as far as we know this is the first report of such a relation between staphylococci and their lytic phage.

In PCSCs phage resistance can be determined genetically and associated with the emergence of phage sensitive cells or it can be determined by phenotypic traits only. At least two phage-dependent mechanisms may be responsible for the latter. In the first case, exemplified by P22 PCSC, proteins that determine superinfection immunity of some cells in the population are inherited cytoplasmically by progeny cells despite the loss of P22 resulting from asymmetric cell division (Cenens et al., 2015). When the phage is removed from the PCSC (e.g., with anti-phage serum) the proportion of cells resistant to superinfection decreases with cell divisions and finally they disappear altogether due to dilution of the immunity factor in the cells. In the second case, uninfected bacteria are modified by soluble factors released from the phage-infected bacteria (Abedon, 2009). For instance lysin released from T7-infected *S. dysenteriae* cells at phage release inactivates the phage receptors of uninfected cells and thus induces a stable equilibrium between infected and non-infected cells (Li et al., 1961). Its concentration decreases with the increasing proportion of modified uninfected cells, until a threshold is reached below which cells recover phage sensitivity (Abedon, 2009). Some bacteria can modify the display of phage receptors or the specificity of their Type I or Type II R-M in the population in a phage-independent manner, e.g., by phase variation (Abedon, 2009; Seed et al., 2012; Cota et al., 2015; Anjum et al., 2016; Aidley et al., 2017; De Ste Croix et al., 2017). The phage population in this case would be maintained solely by infecting those bacteria in the population that currently display the receptor or do not restrict a given phage DNA. Phase variation is also a cause of intra-populational diversity of certain surface properties in *S. aureus*. For instance the adhesive properties and the ability to form biofilm may be diversified among the cells of a population as a result of reversible insertion/excision of IS256 or the expansion/contraction of a tandem repeat in *icaC* – a gene involved in the synthesis of poly-*N*-acetylglucosamine (PNAG) which is a major component of the biofilm matrix (Kiem et al., 2004; Arciola et al., 2015).

The maintenance of PCSCs can be advantageous to bacterial populations. For instance PCSCs of *Campylobacter jejuni* could serve as phage delivery vectors to new target hosts within pre-colonized chicken, facilitating the acquisition of new environment for the bacteria and for its phage (Šišáková et al., 2013). Here, we show that the ability of bacteriophage phiAGO1.3 to be maintained in the form of a phage carrier state population with its host *S. aureus* strain in laboratory cultures does not preclude its ability to cure *C. elegans* from infection with the same *S. aureus* strain. A few reasons for this discrepancy are possible: (i) the phage carrier state could be limited to certain conditions only and cannot be maintained *in vivo*, (ii) limitation of the bacterial load by phages killing the sensitive fraction of cells suffices for the natural antimicrobial defense of the nematode to combat the infection, (iii) the phage carrier state somehow interferes with the bacterial virulence.

In certain bacterial pathogens phase-variation-dependent phage resistance phenotypes are associated with changes that abolish virulence (Seed et al., 2012; Cota et al., 2015). The maintenance of the subpopulation of phage susceptible cells may be a temporal cost overweighted by the increased fitness of these pathogens upon infection of a human or animal. We found here that the mutation emerging in the population of 80wphwpl cells infected with phiAGO1.3 and causing the resistance to phiAGO1.3 leads to the synthesis of a truncated inactive version of ArlS protein. ArlS is a part of the two-component ArlR-ArlS system known to affect several functions associated with *S. aureus* virulence and surface properties, either directly or through other regulatory systems, *agr* among them (Fournier and Hooper, 2000; Fournier et al., 2001; Liang et al., 2005; Luong and Lee, 2006). The ArlRS system is involved in catheter colonization and in endothelial cell damage by *S. aureus* (Burgui et al., 2018; Seidl et al., 2018). It is required for establishing invasive *S. aureus* infection and resistance to calprotectin-mediated Mn starvation *in vivo* and has been shown to be essential for pathogenesis in a rabbit model of sepsis and infective endocarditis (Walker et al., 2013; Radin et al., 2016). Mutations that abolish ArlRS functioning cause, among other defects, the inability of cells to agglutinate, decrease protein A expression in certain media, capsule production and intercellular adhesion, and lead to a serious deficit in the PNAG production (Luong and Lee, 2006; Burgui et al., 2018; Villanueva et al., 2018).

Certain virulence factors that are down-regulated in the ArlRS-deficient mutant, e.g., the Ser-Asp-rich bone fibronectin-binding proteins SdrC, SdrD, and SdrE (Liang et al., 2005) require sortase A (SrtA) to be covalently anchored to the cell wall peptidoglycan (Mazmanian et al., 2001). *S. aureus srtA* deletion mutants have been shown to be resistant to infection with phiAGO1.3-related phages 44AHJD, 66 and 68 (Li et al., 2015). It seems plausible that the cell surface change that causes the phiAGO1.3 resistance of *arlS* mutants concerns a sortase-dependent protein. One of the Sdr proteins is a candidate.

A common feature of the *srtA* deletion mutant studied by Li et al. (2015) and our *arlS* mutant is their sensitivity to a polyvalent *Kayvirus* phage, K and A5W, respectively. Phage K requires only the WTA backbone for adsorption, independently of WTA modifications (Xia et al., 2010, 2011; Li et al., 2015). Another *Kayvirus*, ϕSA012, contains two RBPs of which one, gp103, binds to α-*O*-GlcNAc substituted WTA and the other, gp105, to the WTA backbone (Takeuchi et al., 2016). Of the two RBPs of A5W (gpORF094 [ACB89087] and gpORF96 [ACB89089.1) one is identical with a phage K RBP, while the other is 98% identical to ϕSA012 gp103, implying that phage A5W can use both the WTA backbone and the α-*O-*GlcNAc substituted WTA for adsorption, unlike phiAGO1.3. Thus, phiAGO1.3 appears to be not only a good candidate for a therapeutic use itself but also as a component of therapeutic phage cocktails with A5W and other *Kayvirus* genus phages.

## Author Contributions

AG-R contributed to the study design, the performance of most of the experimental work and data analysis, and significantly contributed to the drafting and revising of the manuscript. AG contributed to the initial experimental work. JE contributed to the selection and characterization of *S. aureus* strains, contributed to the drafting, and revising of the manuscript. JG, KŻ, and RG contributed to the sequencing and assembly of bacterial genomes. AK contributed to the characterization of S. *aureus* strains. JD performed the proteomic analysis. ML participated in the study design and data analysis and interpretation, the supervision of the study, *in silico* sequence analysis, drafting and revising of the manuscript, and preparation of final version of the manuscript.

## Conflict of Interest Statement

AG-R, JE, and MŁ are joint authors of a patent for a method of phage therapeutic activity testing with the use of nematode. AG, AG-R, JG, MŁ, and RG have filed a patent application for *S. aureus* phage propagator strains. The remaining authors declare that the research was conducted in the absence of any commercial or financial relationships that could be construed as a potential conflict of interest.

## Footnotes

1 https://doi.org/10.1101/274100

2 https://github.com/rrwick/Porechop

3 http://www.ncbi.nlm.nih.gov/Complete_Genomes/SignalCensus.html

## References

[B1] Abedon S. T. (2009). “Disambiguating bacteriophage pseudolysogeny: an historical analysis of lysogeny, pseudolysogeny, and the phage carrier state,” in *Contemporary Trends in Bacteriophage Research*, ed. Adams H. T. (New York, NY: Nova Science Publishers), 285–307.

[B2] Abedon S. T. (2017). *Expected Efficacy: Applying Killing Titer Estimations to Phage Therapy Experiments*. Available at: http://www.phage-therapy.org/writings/killing_titers.html

[B3] Aidley J. Sørensen M. C. H. Bayliss C. D. Brøndsted L. (2017). Phage exposure causes dynamic shifts in the expression states of specific phase-variable genes of *Campylobacter jejuni*. *Microbiology* 163 911–919. 10.1099/mic.0.000470 28597819

[B4] Anjum A. Brathwaite K. J. Aidley J. Connerton P. L. Cummings N. J. Parkhill J. (2016). Phase variation of a Type IIG restriction-modification enzyme alters site-specific methylation patterns and gene expression in *Campylobacter jejuni* strain NCTC11168. *Nucleic Acids Res.* 44 4581–4594. 10.1093/nar/gkw019 26786317 PMC4889913

[B5] Arciola C. R. Campoccia D. Ravaioli S. Montanaro L. (2015). Polysaccharide intercellular adhesin in biofilm: structural and regulatory aspects. *Front. Cell Infect. Microbiol.* 5:7. 10.3389/fcimb.2015.00007 25713785 PMC4322838

[B6] Bal A. M. Coombs G. W. Holden M. T. G. Lindsay J. A. Nimmo G. R. Tattevin P. (2016). Genomic insights into the emergence and spread of international clones of healthcare-, community- and livestock-associated meticillin-resistant *Staphylococcus aureus*: blurring of the traditional definitions. *J. Glob. Antimicrob. Resist.* 6 95–101. 10.1016/j.jgar.2016.04.004 27530849

[B7] Barrera-Rivas C. I. Valle-Hurtado N. A. González-Lugo G. M. Baizabal-Aguirre V. M. Bravo-Patiño A. Cajero-Juárez M. (2017). “Bacteriophage therapy: an alternative for the treatment of *Staphylococcus aureus* infections in animals and animal models,” in *Frontiers in Staphylococcus aureus*, eds Enany S. M. E. Crotty-Alexander L. E. (London: IntechOpen), 179–201.

[B8] Barylski J. Enault F. Dutilh B. E. Schuller M. P. B. Edwards R. A. Gillis A. (2018). Analysis of spounaviruses as a case study for the overdue reclassification of tailed bacteriophages. *bioRxiv* [Preprint]. 10.1101/220434 PMC7409376 31127947

[B9] Borysowski J. Łobocka M. B. Międzybrodzki R. Weber-Dabrowska B. Górski A. (2011). Potential of bacteriophages and their lysins in the treatment of MRSA: current status and future perspectives. *BioDrugs* 25 347–355. 10.2165/11595610-000000000-00000 22050337

[B10] Brown S. Xia G. Luhachack L. G. Campbell J. Meredith T. C. Chen C. (2012). Methicillin resistance in *Staphylococcus aureus* requires glycosylated wall teichoic acids. *Proc. Natl. Acad. Sci. U.S.A.* 109 18909–18914. 10.1073/pnas.1209126109 23027967 PMC3503181

[B11] Burgui S. Gil C. Solano C. Lasa I. Valle J. (2018). A systematic evaluation of the two-component systems network reveals that ArlRS is a key regulator of catheter colonization by *Staphylococcus aureus*. *Front. Microbiol.* 9:342. 10.3389/fmicb.2018.00342 29563900 PMC5845881

[B12] Cao L. Gao C. H. Zhu J. Zhao L. Wu Q. Li M. (2016). Identification and functional study of type III-A CRISPR-Cas systems in clinical isolates of *Staphylococcus aureus*. *Int. J. Med. Microbiol.* 306 686–696. 10.1016/j.ijmm.2016.08.005 27600408

[B13] Carlson K. (2004). “Working with bacteriophages: common techniques and methodological approaches,” in *Bacteriophages Biology and Applications*, eds Kutter E. Sulakvelidze A. (Boca Raton, FL: CRC Press), 437–494. 10.1201/9780203491751.ax1

[B14] Cenens W. Makumi A. Govers S. K. Lavigne R. Aertsen A. (2015). Viral transmission dynamics at single-cell resolution reveal Transiently immune subpopulations caused by a carrier state association. *PLoS Genet.* 11:e1005770. 10.1371/journal.pgen.1005770 26720743 PMC4697819

[B15] Chen K. Stephanou A. S. Roberts G. A. White J. H. Cooper L. P. Houston P. J. (2016). The Type I restriction enzymes as barriers to horizontal gene transfer: Determination of the DNA target sequences recognised by livestock-associated methicillin-resistant *Staphylococcus aureus* clonal complexes 133/ST771 and 398. *Adv. Exp. Med. Biol.* 915 81–97. 10.1007/978-3-319-32189-9_7 27193539

[B16] Chen S. Zhou Y. Chen Y. Gu J. (2018). fastp: an ultra-fast all-in-one FASTQ preprocessor. *Bioinformatics* 34 i884–i890. 10.1093/bioinformatics/bty560 30423086 PMC6129281

[B17] Chopin M. C. Chopin A. Bidnenko E. (2005). Phage abortive infection in lactococci: variations on a theme. *Curr. Opin. Microbiol.* 8 473–479. 10.1016/j.mib.2005.06.006 15979388

[B18] Cooper L. P. Roberts G. A. White J. H. Luyten Y. A. Bower E. K. M. Morgan R. D. (2017). DNA target recognition domains in the Type I restriction and modification systems of *Staphylococcus aureus*. *Nucleic Acids Res.* 45 3395–3406. 10.1093/nar/gkx067 28180279 PMC5399793

[B19] Cosgrove S. E. Sakoulas G. Perencevich E. N. Schwaber M. J. Karchmer A. W. Carmeli Y. (2003). Comparison of mortality associated with methicillin-resistant and methicillin-susceptible *Staphylococcus aureus* bacteremia: a meta-analysis. *Clin. Infect. Dis* 36 53–59. 10.1086/345476 12491202

[B20] Cota I. Sánchez-Romero M. A. Hernández S. B. Pucciarelli M. G. García-Del Portillo F. Casadesús J. (2015). Epigenetic control of *Salmonella enterica* O-Antigen Chain Length: a tradeoff between virulence and bacteriophage resistance. *PLoS Genet.* 11:e1005667. 10.1371/journal.pgen.1005667 26583926 PMC4652898

[B21] de Kraker M. E. A. Peter G. Davey P. G. Grundmann H. (2011). Mortality and hospital stay associated with resistant *Staphylococcus aureus* and *Escherichia coli* bacteremia: estimating the burden of antibiotic resistance in Europe. *PLoS Med.* 8:e1001104. 10.1371/journal.pmed.1001104 22022233 PMC3191157

[B22] De Ste Croix M. Vacca I. Kwun M. J. Ralph J. D. Bentley S. D. Haigh R. (2017). Phase-variable methylation and epigenetic regulation by type I restriction-modification systems. *FEMS Microbiol. Rev.* 41(Suppl._1), S3–S15. 10.1093/femsre/fux025 28830092

[B23] Dempsey R. M. Carroll D. Kong H. Higgins L. Keane C. T. Coleman D. C. (2005). Sau42I, a BcgI-like restriction-modification system encoded by the *Staphylococcus aureus* quadruple-converting phage Phi42. *Microbiology* 151 1301–1311. 10.1099/mic.0.27646-0 15817797

[B24] Depardieu F. Didier J. P. Bernheim A. Sherlock A. Molina H. Duclos B. (2016). A eukaryotic-like serine/threonine kinase protects staphylococci against phages. *Cell Host Microbe.* 20 471–481. 10.1016/j.chom.2016.08.010 27667697

[B25] d’Herelle F. (1931a). An address on bacteriophagy and recovery from infectious diseases. *Can. Med. Assoc. J.* 24 619–628. 20318284 PMC382443

[B26] d’Herelle F. (1931b). Bacteriophage as a treatment in acute medical and surgical infections. *Bull. N. Y. Acad. Med.* 7 329–348. 19311785 PMC2095997

[B27] Dreier J. MacWilliams M. P. Bickle T. A. (1996). DNA cleavage by the type IC restriction-modification enzyme EcoR124II. *J. Mol. Biol.* 264 722–733. 10.1006/jmbi.1996.0672 8980681

[B28] Fadlallah A. Chelala E. Legeais J. M. (2015). Corneal infection therapy with topical bacteriophage administration. *Open Ophthalmol. J.* 9 167–168. 10.2174/1874364101509010167 26862360 PMC4740968

[B29] Fish R. Kutter E. Wheat G. Blasdel B. Kutateladze M. Kuhl S. (2016). Bacteriophage treatment of intransigent diabetic toe ulcers: a case series. *J. Wound Care* 25(Suppl. 7), S27–S33. 10.12968/jowc.2016.25.7.S2726949862

[B30] Fournier B. Hooper D. C. (2000). A new two-component regulatory system involved in adhesion, autolysis, and extracellular proteolytic activity of *Staphylococcus aureus*. *J. Bacteriol.* 182 3955–3964. 10.1128/JB.182.14.3955-3964.2000 10869073 PMC94580

[B31] Fournier B. Klier A. Rapoport G. (2001). The two-component system ArlS-ArlR is a regulator of virulence gene expression in *Staphylococcus aureus*. *Mol. Microbiol.* 41 247–261. 10.1046/j.1365-2958.2001.02515.x 11454217

[B32] Godány A. Bukovská G. Farkasovská J. Brnáková Z. Dmitriev A. Tkáciková E. (2004). Characterization of a complex restriction-modification system detected in *Staphylococcus aureus* and *Streptococcus agalactiae* strains isolated from infections of domestic animals. *Folia Microbiol.* 49 307–314. 10.1007/BF02931048 15259773

[B33] Goerke C. Pantůček R. Holtfreter S. Schulte B. Zink M. Grumann D. (2009). Diversity of prophages in dominant *Staphylococcus aureus* clonal lineages. *J. Bacteriol.* 191 3462–3468. 10.1128/JB.01804-08 19329640 PMC2681900

[B34] Golec P. Dąbrowski K. Hejnowicz M. S. Gozdek A. Łoś J. M. Wegrzyn G. (2011). A reliable method for storage of tailed phages. *J. Microbiol. Methods* 84 486–489. 10.1016/j.mimet.2011.01.007 21256885

[B35] Gozdek A. Głowacka-Rutkowska A. Gawor J. Empel J. Gromadka R. Łobocka M. B. (2018). Complete genome sequences of two novel *Staphylococcus aureus* podoviruses of potential therapeutic use, vB_SauP_phiAGO1.3 and vB_SauP_phiAGO1.9. *Genome Announc.* 6 4–5. 10.1128/genomeA.00048-18 29700131 PMC5920172

[B36] Hooton S. P. Brathwaite K. J. Connerton I. F. (2016). The bacteriophage carrier state of *Campylobacter jejuni* features changes in host non-coding RNAs and the acquisition of new host-derived CRISPR spacer sequences. *Front. Microbiol.* 7:355. 10.3389/fmicb.2016.00355 27047470 PMC4804229

[B37] Ito T. Ma X. X. Takeuchi F. Okuma K. Yuzawa H. Hiramatsu K. (2004). Novel type V staphylococcal cassette chromosome *mec* driven by a novel cassette chromosome recombinase, *ccrC*. *Antimicrob. Agents Chemother.* 48 2637–2651. 10.1128/AAC.48.7.2637-2651.2004 15215121 PMC434217

[B38] Iwano H. Inoue Y. Takasago T. Kobayashi H. Furusawa T. Taniguchi K. (2018). Bacteriophage ΦSA012 has a broad host range against *Staphylococcus aureus* and effective lytic capacity in a mouse mastitis model. *Biology* 7:E8. 10.3390/biology7010008 29315249 PMC5872034

[B39] Iyer L. M. Burroughs A. M. Anand S. de Souza R. F. Aravind L. (2017). Polyvalent proteins, a pervasive theme in the intergenomic biological conflicts of bacteriophages and conjugative elements. *J. Bacteriol* 199:e00245-17. 10.1128/JB.00245-17 28559295 PMC5512222

[B40] Jindrova E. Schmid-Nuoffer S. Hamburger F. Janscak P. Bickle T. A. (2005). On the DNA cleavage mechanism of Type I restriction enzymes. *Nucleic Acids Res.* 33 1760–1766. 10.1093/nar/gki322 15788748 PMC1069518

[B41] Kaźmierczak Z. Górski A. Dąbrowska K. (2014). Facing antibiotic resistance: *Staphylococcus aureus* phages as a medical tool. *Viruses* 6 2551–2570. 10.3390/v6072551 24988520 PMC4113783

[B42] Kiem S. Oh W. S. Peck K. R. Lee N. Y. Lee J. Y. Song J. H. (2004). Phase variation of biofilm formation in *Staphylococcus aureus* by IS 256 insertion and its impact on the capacity adhering to polyurethane surface. *J. Korean Med. Sci.* 19 779–782. 10.3346/jkms.2004.19.6.779 15608385 PMC2816298

[B43] Kraushaar B. Thanh M. D. Hammerl J. A. Reetz J. Fetsch A. Hertwig S. (2013). Isolation and characterization of phages with lytic activity against methicillin-resistant *Staphylococcus aureus* strains belonging to clonal complex 398. *Arch. Virol.* 158 2341–2350. 10.1007/s00705-013-1707-6 23760627

[B44] Kreiswirth B. N. Löfdahl S. Betley M. J. O’Reilly M. Schlievert P. M. Bergdoll M. S. (1983). The toxic shock syndrome exotoxin structural gene is not detectably transmitted by a prophage. *Nature* 305 709–712. 10.1038/305709a0 6226876

[B45] Kwan T. Liu J. DuBow M. Gros P. Pelletier J. (2005). The complete genomes and proteomes of 27 *Staphylococcus aureus* bacteriophages. *Proc. Natl. Acad. Sci. U.S.A.* 102 5174–5179. 10.1073/pnas.0501140102 15788529 PMC556006

[B46] Labrie S. J. Samson J. E. Moineau S. (2010). Bacteriophage resistance mechanisms. *Nat. Rev. Microbiol.* 8 317–327. 10.1038/nrmicro2315 20348932

[B47] Lavigne R. Seto D. Mahadevan P. Ackermann H. W. Kropinski A. M. (2008). Unifying classical and molecular taxonomic classification: analysis of the Podoviridae using BLASTP-based tools. *Res. Microbiol.* 159 406–414. 10.1016/j.resmic.2008.03.005 18555669

[B48] Li K. Barksdale L. Garmise L. (1961). Phenotypic alterations associated with the bacteriophage carrier state of *Shigella* dysenteriae. *J. Gen. Microbiol.* 24 355–367. 10.1099/00221287-24-3-355 13761818

[B49] Li X. Gerlach D. Du X. Larsen J. Stegger M. Kühner P. (2015). An accessory wall teichoic acid glycosyltransferase protects *Staphylococcus aureus* from the lytic activity of Podoviridae. *Sci Rep.* 5:17219. 10.1038/srep17219 26596631 PMC4667565

[B50] Liang X. Zheng L. Landwehr C. Lunsford D. Holmes D. Ji Y. (2005). Global regulation of gene expression by ArlRS, a two-component signal transduction regulatory system of *Staphylococcus aureus*. *J. Bacteriol.* 187 5486–5492. 10.1128/JB.187.15.5486-5492.2005 16030243 PMC1196029

[B51] Lindsay J. A. (2014). *Staphylococcus aureus* genomics and the impact of horizontal gene transfer. *Intl. J. Med. Microbiol.* 304 103–109. 10.1016/j.ijmm.2013.11.010 24439196

[B52] Łobocka M. Hejnowicz M. S. Gągała U. Weber-Dąbrowska B. Wêgrzyn G. Dadlez M. (2014a). “The First Step to Bacteriophage Therapy - How to Choose the Correct Phage,” in *Phage Therapy: Current Research and Applications*, eds Borysowski J. Międzybrodzki R. Górski A. (Poole: Caister Academic Press), 23–69.

[B53] Łobocka M. B. Głowacka A. Dąbrowski K. Hejnowicz M. S. Gozdek A. Weber-Dąbrowska B. (2014b). *A Method of Evaluating the Therapeutic Efficacy of Bacteriophages*. *Pat. UPRP PL*219654 B1, Pat. EP2872156 B1; Pat. US 9678063 B2, WO2014/012872 A1.

[B54] Łobocka M. B. Hejnowicz M. S. Dąbrowski K. Gozdek A. Kosakowski J. Witkowska M. (2012). Genomics of staphylococcal Twort-like phages: potential therapeutics of the post-antibiotic era. *Adv. Virus Res.* 83 143–216. 10.1016/B978-0-12-394438-2.00005-0 22748811

[B55] Łobocka M. B. Hejnowicz M. S. Dąbrowski K. Izak D. Gozdek A. Głowacka A. (2016). *Staphylococcus aureus* strains for the production of monoclonal bacteriophage preparations deprived of plasmid DNA. WO 2016/030871 A1. U.S. Patent. 2016 Mar 16.

[B56] Luong T. T. Lee C. Y. (2006). The arl locus positively regulates *Staphylococcus aureus* type 5 capsule via an mgrA-dependent pathway. *Microbiology* 152 3123–3131. 10.1099/mic.0.29177-0 17005991

[B57] Lwoff A. (1953). Lysogeny. *Bacteriol. Rev.* 17 269–337. 13105613 10.1128/br.17.4.269-337.1953 PMC180777

[B58] MacNeal W. J. Frisbee F. C. (1936a). Bacteriophage service to patients with Staphylococcus septicemia. *Am. J. Med. Sci.* 191 170–178. 10.1097/00000441-193602000-00003

[B59] MacNeal W. J. Frisbee F. C. (1936b). One hundred patients with *Staphylococcus septicemia* receiving bacteriophage service. *Am. J. Med. Sci.* 191 179–195. 10.1097/00000441-193602000-00004

[B60] Mandell G. L. Bennett J. E. Dolin R. (2010). *Mandell, Douglas, and Bennett’s Principles and Practice of Infectious Diseases, Churchill Livingstone*. Philadelphia, PA: Elsevier.

[B61] Mazmanian S. K. Ton-That H. Schneewind O. (2001). Sortase-catalysed anchoring of surface proteins to the cell wall of *Staphylococcus aureus*. *Mol. Microbiol.* 40 1049–1057. 10.1046/j.1365-2958.2001.02411.x 11401711

[B62] McCarthy A. J. Lindsay J. A. (2010). Genetic variation in *Staphylococcus aureus* surface and immune evasion genes is lineage associated: implications for vaccine design and host-pathogen interactions. *BMC Microbiol.* 10:173. 10.1186/1471-2180-10-173 20550675 PMC2905362

[B63] McCarthy A. J. Lindsay J. A. (2012). The distribution of plasmids that carry virulence and resistance genes in *Staphylococcus aureus* is lineage associated. *BMC Microbiol.* 12:104. 10.1186/1471-2180-12-104 22691167 PMC3406946

[B64] McCarthy A. J. Witney A. A. Lindsay J. A. (2012). *Staphylococcus aureus* temperate bacteriophage: carriage and horizontal gene transfer (HGT) is lineage associated. *Front. Cell Infect. Microbiol.* 2:6 10.3389/fcimb.2012.00006 PMC3417521 22919598

[B65] Międzybrodzki R. Borysowski J. Weber-Dąbrowska B. Fortuna W. Letkiewicz S. Szufnarowski K. (2012). Clinical aspects of phage therapy. *Adv. Virus Res.* 83 73–121. 10.1016/B978-0-12-394438-2.00003-7 22748809

[B66] Monk I. R. Shah I. M. Xu M. Tan M. W. Foster T. J. (2012). Transforming the untransformable: application of direct transformation to manipulate genetically *Staphylococcus aureus* and *Staphylococcus epidermidis*. *mBio* 3:e00277-11. 10.1128/mBio.00277-11 22434850 PMC3312211

[B67] Monk I. R. Tree J. J. Howden B. P. Stinear T. P. Foster T. J. (2015). Complete bypass of restriction systems for major *Staphylococcus aureus* lineages. *mBio* 6:e00308-15. 10.1128/mBio.00308-15 26015493 PMC4447248

[B68] Moormeier D. E. Bayles K. W. (2017). *Staphylococcus aureus* biofilm: a complex developmental organism. *Mol. Microbiol.* 104 365–376. 10.1111/mmi.13634 28142193 PMC5397344

[B69] Nair D. Memmi G. Hernandez D. Bard J. Beaume M. Gill S. (2011). Whole-genome sequencing of *Staphylococcus aureus* strain RN4220, a key laboratory strain used in virulence research, identifies mutations that affect not only virulence factors but also the fitness of the strain. *J. Bacteriol.* 193 2332–2335. 10.1128/JB.00027-11 21378186 PMC3133102

[B70] Noto M. J. Kreiswirth B. N. Monk A. B. Archer G. L. (2008). Gene acquisition at the insertion site for SCCmec, the genomic island conferring methicillin resistance in *Staphylococcus aureus*. *J. Bacteriol.* 190 1276–1283. 10.1128/JB.01128-07 18083809 PMC2238224

[B71] Otto M. (2012). MRSA virulence and spread. *Cell Microbiol* 14 1513–1521. 10.1111/j.1462-5822.2012.01832.x 22747834 PMC3443268

[B72] Pantůček R. Rosypalová A. Doskar J. Kailerová J. Růzicková V. Borecká P. (1998). The polyvalent staphylococcal phage phi 812: its host-range mutants and related phages. *Virology* 246 241–252. 10.1006/viro.1998.9203 9657943

[B73] Radin J. N. Kelliher J. L. Párraga Solórzano P. K. Kehl-Fie T. E. (2016). The two-component system ArlRS and alterations in metabolism enable *Staphylococcus aureus* to resist calprotectin-induced manganese starvation. *PLoS Pathog.* 12:e1006040. 10.1371/journal.ppat.1006040 27902777 PMC5130280

[B74] Roberts G. A. Houston P. J. White J. H. Chen K. Stephanou A. S. Cooper L. P. (2013). Impact of target site distribution for Type I restriction enzymes on the evolution of methicillin-resistant *Staphylococcus aureus* (MRSA) populations. *Nucleic Acids Res.* 41 7472–7484. 10.1093/nar/gkt535 23771140 PMC3753647

[B75] Samson J. E. Magadán A. H. Sabri M. Moineau S. (2013). Revenge of the phages: defeating bacterial defences. *Nat. Rev. Microbiol.* 11 675–687. 10.1038/nrmicro3096 23979432

[B76] Sauve L. (1936). Le bacteìriophage in chirurgie. *La Med.* 17 49–54. 10.1371/journal.ppat.1002917 23028317 PMC3441752

[B77] Seed K. D. Faruque S. M. Mekalanos J. J. Calderwood S. B. Qadri F. Camilli A. (2012). Phase variable O antigen biosynthetic genes control expression of the major protective antigen and bacteriophage receptor in *Vibrio cholerae* O1. *PLoS Pathog.* 8:e1002917. 10.1371/journal.ppat.1002917 23028317 PMC3441752

[B78] Seidl K. Leemann M. Zinkernagel A. S. (2018). The ArlRS two-component system is a regulator of *Staphylococcus aureus*-induced endothelial cell damage. *Eur. J. Clin. Microbiol. Infect. Dis.* 37 289–292. 10.1007/s10096-017-3130-5 29177635

[B79] Siringan P. Connerton P. L. Cummings N. J. Connerton I. F. (2014). Alternative bacteriophage life cycles: the carrier state of *Campylobacter jejuni*. *Open Biol.* 4:130200. 10.1098/rsob.130200 24671947 PMC3971406

[B80] Šišáková E. van Aelst K. Diffin F. M. Szczelkun M. D. (2013). The type ISP restriction-modification enzymes LlaBIII and LlaGI use a translocation-collision mechanism to cleave non-specific DNA distant from their recognition sites. *Nucleic Acids Res.* 41 1071–1080. 10.1093/nar/gks1209 23222132 PMC3553950

[B81] Son J. S. Lee S. J. Jun S. Y. Yoon S. J. Kang S. H. Paik H. R. (2010). Antibacterial and biofilm removal activity of a Podoviridae *Staphylococcus aureus* bacteriophage SAP-2 and a derived recombinant cell-wall-degrading enzyme. *Appl. Microbiol. Biotechnol.* 86 1439–1449. 10.1007/s00253-009-2386-9 20013118

[B82] Springer B. Orendi U. Much P. Höger G. Ruppitsch W. Krziwanek K. (2009). Methicillin-resistant *Staphylococcus aureus*: a new zoonotic agent? *Wien. Klein. Wochenschr.* 121 86–90. 10.1007/s00508-008-1126-y 19280131

[B83] Stepanovic S. Vukovic D. Dakic I. Savic B. Svabic-Vlahovic M. (2000). A modified microtiter-plate test for quantification of staphylococcal biofilm formation. *J. Microbiol. Methods* 40 175–179. 10.1016/S0167-7012(00)00122-6 10699673

[B84] Stobberingh E. E. Schiphof R. Sussenbach J. S. (1977). Occurrence of a class II restriction endonuclease in *Staphylococcus aureus*. *J. Bacteriol.* 131 645–649. 885840 10.1128/jb.131.2.645-649.1977 PMC235474

[B85] Studier F. W. Bandyopadhyay P. K. (1988). Model for how type I restriction enzymes select cleavage sites in DNA. *Proc. Natl. Acad. Sci. U.S.A.* 85 4677–4681. 2838843 10.1073/pnas.85.13.4677 PMC280498

[B86] Štveráková D. Šedo O. Benešík M. Zdráhal Z. Doškař J. Pantůček R. (2018). Rapid identification of intact staphylococcal bacteriophages using matrix-assisted laser desorption ionization-time-of-flight mass spectrometry. *Viruses* 10 1–19. 10.3390/v10040176 29617332 PMC5923470

[B87] Sulston J. E. Hodgkin J. (1988). “Methods,” in *The Nematode Caenorhabditis elegans*, ed. Wood W. B. (Cold Spring Harbor, NY: Cold Spring Harbor Laboratory Press), 587–606.

[B88] Sussenbach J. S. Monfoort C. H. Schiphof R. Stobberingh E. E. (1976). A restriction endonuclease from *Staphylococcus aureus*. *Nucleic Acids Res.* 3 3193–3202. 10.1093/nar/3.11.3193 1005115 PMC343162

[B89] Sussenbach J. S. Steenbergh P. H. Rost J. A. van Leeuwen W. J. van Embden J. D. (1978). A second site-specific restriction endonuclease from Staphylococcus aureus. *Nucleic Acids Res.* 5 1153–1163. 10.1093/nar/5.4.1153 652518 PMC342067

[B90] Swift S. M. Nelson D. C. (2014). Complete genome sequence of *Staphylococcus aureus* phage GRCS. *Genome Announc.* 2:e00209-14. 10.1128/genomeA.00209-14 24723702 PMC3983291

[B91] Szilák L. Venetianer P. Kiss A. (1990). Cloning and nucleotide sequence of the genes coding for the Sau96I restriction and modification enzymes. *Nucleic Acids Res.* 18 4659–4664. 10.1093/nar/18.16.4659 2204026 PMC331911

[B92] Takemura-Uchiyama I. Uchiyama J. Kato S. Inoue T. Ujihara T. Ohara N. (2013). Evaluating efficacy of bacteriophage therapy against *Staphylococcus aureus* infections using a silkworm larval infection model. *FEMS Microbiol. Lett.* 347 52–60. 10.1111/1574-6968.12220 23869440

[B93] Takemura-Uchiyama I. Uchiyama J. Osanai M. Morimoto N. Asagiri T. Ujihara T. (2014). Experimental phage therapy against lethal lung-derived septicemia caused by *Staphylococcus aureus* in mice. *Microbes Infect.* 16 512–517. 10.1016/j.micinf.2014.02.011 24631574

[B94] Takeuchi I. Osada K. Azam A. H. Asakawa H. Miyanaga K. Tanji Y. (2016). The presence of two receptor-binding proteins contributes to the wide host range of staphylococcal Twort-like phages. *Appl. Environ. Microbiol.* 82 5763–5774. 10.1128/AEM.01385-16 27422842 PMC5038044

[B95] Uchiyama J. Takemura-Uchiyama I. Kato S. Sato M. Ujihara T. Matsui H. (2014). In silico analysis of AHJD-like viruses, *Staphylococcus aureus* phages S24-1 and S13’, and study of phage S24-1 adsorption. *Microbiologyopen* 3 257–270. 10.1002/mbo3.166 24591378 PMC3996573

[B96] Uchiyama J. Taniguchi M. Kurokawa K. Takemura-Uchiyama I. Ujihara T. Shimakura H. (2017). Adsorption of Staphylococcus viruses S13’ and S24-1 on *Staphylococcus aureus* strains with different glycosidic linkage patterns of wall teichoic acids. *J. Gen. Virol.* 98 2171–2180. 10.1099/jgv.0.000865 28730979

[B97] Villanueva M. García B. Valle J. Rapún B. Ruiz de Los Mozos I. Solano C. (2018). Sensory deprivation in *Staphylococcus aureus*. *Nat. Commun.* 9:523. 10.1038/s41467-018-02949-y 29410457 PMC5802764

[B98] Vybiral D. Takác M. Loessner M. Witte A. von Ahsen U. Bläsi U. (2003). Complete nucleotide sequence and molecular characterization of two lytic *Staphylococcus aureus* phages: 44AHJD and P68. *FEMS Microbiol. Lett.* 219 275–283. 10.1016/S0378-1097(03)00028-4 12620632

[B99] Waldron D. E. Lindsay J. A. (2006). Sau1: a novel lineage-specific type I restriction-modification system that blocks horizontal gene transfer into *Staphylococcus aureus* and between *S. aureus* isolates of different lineages. *J. Bacteriol.* 188 5578–5585. 10.1128/JB.00418-06 16855248 PMC1540015

[B100] Walker J. N. Crosby H. A. Spaulding A. R. Salgado-Pabón W. Malone C. L. Rosenthal C. B. (2013). The *Staphylococcus aureus* ArlRS two-component system is a novel regulator of agglutination and pathogenesis. *PLoS Pathog.* 9:e1003819. 10.1371/journal.ppat.1003819 24367264 PMC3868527

[B101] Wang Z. Zheng P. Ji W. Fu Q. Wang H. Yan Y. (2016). SLPW: A virulent bacteriophage targeting methicillin-resistant *Staphylococcus aureus* in vitro and in vivo. *Front. Microbiol.* 7:934. 10.3389/fmicb.2016.00934 27379064 PMC4908117

[B102] West A. H. Stock A. M. (2001). Histidine kinases and response regulator proteins in two-component signaling systems. *Trends Biochem. Sci.* 26 369–376. 10.1016/S0968-0004(01)01852-7 11406410

[B103] Wick R. R. Judd L. M. Gorrie C. L. Holt K. E. (2017). Unicycler: resolving bacterial genome assemblies from short and long sequencing reads. *PLoS Comput. Biol.* 13:e1005595. 10.1371/journal.pcbi.1005595 28594827 PMC5481147

[B104] Xia G. Corrigan R. M. Winstel V. Goerke C. Gründling A. Peschel A. (2011). Wall teichoic acid-dependent adsorption of staphylococcal siphovirus and myovirus. *J. Bacteriol.* 193 4006–4009. 10.1128/JB.01412-10 21642458 PMC3147540

[B105] Xia G. Maier L. Sanchez-Carballo P. Li M. Otto M. Holst O. (2010). Glycosylation of wall teichoic acid in *Staphylococcus aureus* by TarM. *J. Biol. Chem.* 285 13405–13415. 10.1074/jbc.M109.096172 20185825 PMC2859500

[B106] Yang S. Liu J. Shao F. Wang P. Duan G. Yang H. (2015). Analysis of the features of 45 identified CRISPR loci in 32 *Staphylococcus aureus*. *Biochem. Biophys. Res. Commun.* 464 894–900. 10.1016/j.bbrc.2015.07.062 26188514

