# Supplementary material for: The Ability of Lytic Staphylococcal Podovirus vB_SauP_phiAGO1.3 to Coexist in Equilibrium With Its Host Facilitates the Selection of Host Mutants of Attenuated Virulence but Does Not Preclude the Phage Antistaphylococcal Activity in a Nematode Infection Model
Source: Front Microbiol. 2019 Jan 18;9:3227. doi: 10.3389/fmicb.2018.03227 (PMC6346686; doi:10.3389/fmicb.2018.03227)
Supplement: Supplementary file 1 [file Data_Sheet_1.pdf]

Supplementary Material

The ability of a lytic staphylococcal podovirus vB_SauP_phiAGO1.3 to coexist in an equilibrium with its host facilitates the selection of host mutants of attenuated virulence but does not preclude the phage antistaphylococcal activity in a nematode infection model

Aleksandra Głowacka-Rutkowska[1], Agnieszka Gozdek[1], Joanna Empel[2], Jan Gawor[3], Karolina Żuchniewicz[3], Aleksandra Kozińska[2#], Janusz Dębski[4], Robert Gromadka[3], Małgorzata Łobocka[1, 5]*

[1]Department of Microbial Biochemistry, Institute of Biochemistry and Biophysics, Polish Academy of Sciences, Warsaw, Poland

[2]Department of Epidemiology and Clinical Microbiology, National Medicines Insitute (NMI), Warsaw. Poland

[3]Laboratory of DNA Sequencing and Oligonucleotide Synthesis, Institute of Biochemistry and Biophysics , Polish Academy of Sciences, Warsaw, Poland

[4]Laboratory of Mass Spectrometry, Institute of Biochemistry and Biophysics , Polish Academy of Sciences, Warsaw, Poland

[5]Autonomous Department of Microbial Biology, Faculty of Agriculture and Biology, Warsaw University of Life Sciences, Warsaw, Poland

[#] Present address: Department of Drug Biotechnology and Bioinformatics, NMI

*** Correspondence: Małgorzata Łobocka: lobocka@ibb.waw.pl**

1 Supplementary experimental procedures

1.1 Determination of the optimal multiplicity of infection

Overnight culture of 80wphwpl strain was diluted 1:100 in fresh LB and incubated at 37°C with shaking (200 rpm) until the optical density (OD_{600}) of about 0.4. Cells were mixed with phages at different MOIs (100; 10; 1; 0.1; 0.01; 0.001 and 0.0001), left for 15 min at room temperature (RT) without shaking to allow for phage adsorption and incubated for 3.5 h at 37°C with shaking (200 rpm). Next, the cultures were centrifuged (10,000 x g, 10 min, 4ºC). Supernatants were filtered

through 0.22 µm pore size membranes (MILLEX® GS), immediately diluted, and then titers of phage were determined by the double-layered-agar plate method according to Adams (1959).

1.2 Phage adsorption

Phage adsorption experiments were carried out as described previously (Shao and Wang, 2008) with some modifications. Culture of 80wphwpl strain (OD_{600} of about 0.4) was infected with phiAGO1.3 at MOI of 0.1 and the mixture was incubated at RT. Samples (1 ml) were taken after 2, 4, 6, 8, 10 and 15 minutes and immediately centrifuged (10,000 x g, 2 min). Supernatants containing unadsorbed phages were filtered through 0.22 µm pore size membranes (MILLEX® GS). Phage titers were then determined by the double-layer agar plate method.

1.1 Determination of phage latent period and burst size

One-step growth experiments were performed to determine the latent period and burst size of phiAGO1.3. Briefly, cells of 80wphwpl culture (10 ml, OD_{600} of about 0.4) were harvested by centrifugation (10,000 × *g* for 5 min) and resuspended in 10 ml of fresh LB. Phages were added at MOI of about 0.1 and allowed to adsorb for 10 min at 37°C. The mixture was centrifuged (10,000 x g, 4 min, RT), resuspended in 10 ml of fresh, prewarmed (37°C) LB and incubated at 37°C with shaking (200 rpm). Samples were taken at 2-min intervals (up to 60 min), immediately diluted, and then titers of phage were determined by the double-layer agar plate method.

1.2 Determination of phage stability at different temperatures and different pH

For thermostability testing, 1 ml of phage-containing lysate (10^7 PFU/ml) was incubated at different temperatures (37, 50, 60, 70°C) for 5, 15, 40, 60 min, and titrated immediately using the double-layer agar plate method. For phage stability testing at different pH, phage-containing lysate (10^9 PFU/ml) was diluted 1:100 in LB medium of pH ranging from 3 to 11 (adjusted with 1 M HCl or 1 M NaOH) incubated for 1 h at 37°C, and titrated using the double-layer agar plate method.

1.5 Identification of virion proteins

Phage suspension (10^{11} PFU/ml) in phosphate buffer saline (PBS: 10 mM Na_2HPO_4, 137 mM NaCl, 2.7 mM KCl, 2 mM KH_2PO_4 at pH 7.4). was loaded on a block gradient of CsCl (CsCl densities: 1.3, 1.5, 1.7 g/ml in PBS) and centrifuged in TH-641 Swinging Bucket Rotor (Thermo Scientific) at 22 000 rpm, at 4°C for 2 h. The band located between CsCl densities 1.3 and 1.5 g/ml was collected and used as a source of virion proteins. Peptides of whole phiAGO1.3 virions were identified at the Laboratory of Mass Spectrometry of IBB PAS, following tryptic digestion of phiAGO1.3 virion proteins as described elsewhere (Szczepankowska et al., 2017). The analysis was performed with virions from two independently prepared samples. Predicted peptides of all six ORFs of phiAGO1.3 genome were used to search for matching hits.

2 Supplementary Figures and Tables

2.1 Tables

Supplementary Table 1. *Staphylococcus* sp. strains other than *S. aureus* used in this study. Collection numbers of deposits, used to determine the genomic sequence of particular strain are bolded.

***Staphylococcus* species**	**DSM no.**	**Other collection no.**	**Coagulase**	**NCBI acc. no.**
Staphylococcus delphini	20771	ATCC 49171	plus	
Staphylococcus intermedius	20373	ATCC 29663, CCM 5739, **NCTC 11048**	plus	UHDP00000000.1
Staphylococcus pseudintermedius	21284	**CCUG 49543**, CIP 108864, LMG 22219	plus	NZ_PPRB00000000.1
Staphylococcus hyicus	17421	CIP 64.51	plus	
Staphylococcus hyicus	20459	**ATCC 11249**, CCM 2368, NCTC 10350	plus	NZ_CP008747.1
Staphylococcus saprophyticus subsp. bovis	18669	CCM 4410, CCUG 36975, **CCUG 38042**, CIP 105260	plus	NZ_PPRA00000000.1
Staphylococcus saprophyticus subsp. saprophyticus	20229	ATCC **15305**, CCM 883, NCIB 8711, NCTC 7292, WDCM 00159	plus	NZ_MTGA00000000.1
Staphylococcus schleiferi subsp. coagulans	**6628**	ATCC 49545, CIP 104370, JCM 7470	plus	NZ_PPQN00000000.1
Staphylococcus lugdunensis	4804	ATCC 43809	minus	
Staphylococcus epidermidis	20044	**ATCC 14990**, CCM 2124, WDCM [illegible]	minus	NZ_NARC00000000.1
Staphylococcus caprae	20608	ATCC 35538, CCM 3573	minus	
Staphylococcus chromogenes	20454	ATCC 43764, CCM 3387, **NCTC 10530**	minus	NZ_PPQK00000000.1

Supplementary Table 2. Homologies of phiAGO1.3 proteins to proteins of related phages infecting *S. aureus* (% coverage/% identity). The closest homologs of respective phiAGO1.3 proteins are highlighted. The NCBI accession numbers of phage genomic sequences are the following: NC_004678.1 (44AHJD), NC_023550.1 (GRCS), AY954949.1 (66), NC_009875.1 (SAP-2), AB626963.1 (S13'), NC_031046.1 (BP39), NC_031008.1 (SLPW), KY000084.1 (SCH1), NC_016565.1 (S24-1), AF513033.1 (68), HF937074.1 (PSa3). Phage phiAGO1.9, which is nearly identical to phiAGO1.3 (Gozdek et al., 2018) and phage SCH111 (GenBank acc. no. KY000084), which differs from SCH1 only by 3 nucleotide residues are not included. Phages St 134 and Andhra, which infect *S. epidermidis* and are more distant relatives of phiAGO1.3 than other phages in the Table, are not included. Virion proteins are marked by an asterisk (see Supplementary Table 3). Names of representative subgroup I phages are underlined.

**phiAGO1.3 ORF no.**	**Known or predicted function**	**Phage**										
		**44AHJD**	**GRCS**	**66**	**SAP-2**	**S13'**	**BP39**	**SLPW**	**SCH1**	**S24-1**	**68**	**PSa3**
1*		100/96	100/96	100/96	100/95	100/95	100/97	100/97	100/96	100/96	100/97	100/92
2	100/100	87/100	81/100	87/100	77/100	88/100	95/100	90/100	94/100	95/100	86/100	79/100
3		97/100	93/100	96/100	94/100	97/100	96/100	95/100	98/100	98/98	98/100	97/100
4		97/100	86/100	100/100	42/100	91/100	42/100	42/100	42/100	90/100	97/100	98/100
5		51/100	67/100	77/100	85/100	75/100	42/100	-	83/100	73/100	51/100	-
6		93/100	93/100	93/100	85/100	73/100	89/100	87/100	91/100	89/100	93/100	93/100
7*		94/100	76/95	93/100	76/95	96/100	96/100	54/100| 100//26	96/100	97/100	93/100	95/95
8		97/91	92/100	97/100	99/91		99/100	98/100	99/100	98/100		98/100
9		96/99	96/100	95/100	97/100	95/100	95/99	97/100	97/100	96/100	96/99	96/100
10*	PlyCA, tail lysin	93/100	96/100	93/100	96/100	96/100	76/100	92/100	96/100	96/100	93/100	92/100
11	holin	93/100	98/100	93/100	93/100	98/100	95/100	98/100	100/38	99/100	93/100	96/100
12*	tail-knob	98/100	99/100	97/100	97/100	98/100	97/100	98/100	98/100	98/100	98/100	98/100
13*	tail fiber	-	84/100	81/42| 31/57	80/57	63/100	76/100	77/42| 33/57	65/100	54/100	65/100	57/100
14	amidase	90/100	91/100	90/100	92/100	88/100	91/100	89/100	82/100	88/100	90/100	95/100
15*	RBP	96/100	96/100	96/100	94/100	95/99	68/100	95/99	94/100	69/100	96/100	96/100
16*	lower collar	97/100	98/100	97/100	94/98	94/100	94/100	95/100	94/100	94/100	97/100	98/100
17*	uper collar	93/76	100/100	99/100	98/76	93/100	98/100	93/100	93/100	93/100	93/79	99/100
18*	capsid	95/100	96/100	95/100	97/100	91/98	94/98	91/98	91/98	91/98	95/100	98/100
19*		95/100	98/100	95/100	98/100	95/100	95/100	97/100	95/100	95/100	95/100	100/100
20*		84/100	86/100	81/100	83/100	82/100	80/100	72/100	65/100	78/100	84/100	93/100

Supplementary Table 3. Virion proteins of phiAGO1.3 identified by mass spectrometry after tryptic digestion of whole virions

Protein ID	ORF #	ORF start	ORF end	Strand	No. of AA residues	M.W. kDa	No. of matching peptides	Sequence coverage (%)	Mascot score	pI	Known/predicted function	44AHJD (Identity in %)
AUS03367.1	1	445	747	+	100	11.61	1/1	10/9	37/37	4.04	Hypothetical protein	ORF2 (96)
AUS03373.1	7	2202	2684	+	160	19.48	29/31	61/47	977/935	7.08	Hypothetical protein	ORF8 (94)
AUS03376.1	10	6394	7824	-	476	51.98	51/63	31/31	1874/2169	8.31	Tail lysin	ORF11 (93)
AUS03378.1	12	8223	9986	-	587	68.50	40/40	19/19	1439/1326	5.74	Major tail protein	ORF13 (98)
AUS03379.1	13	10042	10938	-	298	33.93	72/86	47/58	2710/2845	5.32	Minor tail protein	absent
AUS03381.1	15	11767	13704	-	645	74.45	80/94	37/39	3143/3234	5.33	Receptor bindin protein	ORF17 (96)
AUS03382.1	16	13718	14473	-	251	29.17	7/7	5/5	222/200	5.03	Lower collar protein	ORF18 (97)
AUS03383.1	17	14466	15449	-	327	37.91	44/45	23/23	1630/1547	4.99	Upper collar protein	ORF19 (99)
AUS03384.1	18	15464	16690	-	408	46.92	76/87	46/50	3388/3512	5.35	Major capsid protein	ORF20 (95)
AUS03385.1	19	16697	16879	-	59	6.81	69/90	83/100	3676/4108	4.38	Hypothetical protein	ORF21 (95)
AUS03386.1	20	16892	17263	-	123	14.16	60/112	51/60	2863/4596	3.62	Hypothetical protein	ORF22 (84)

*Two independent samples were used for the analysis. Values obtained with each of the two samples are separated by "/".

2.2 Supplementary Figures

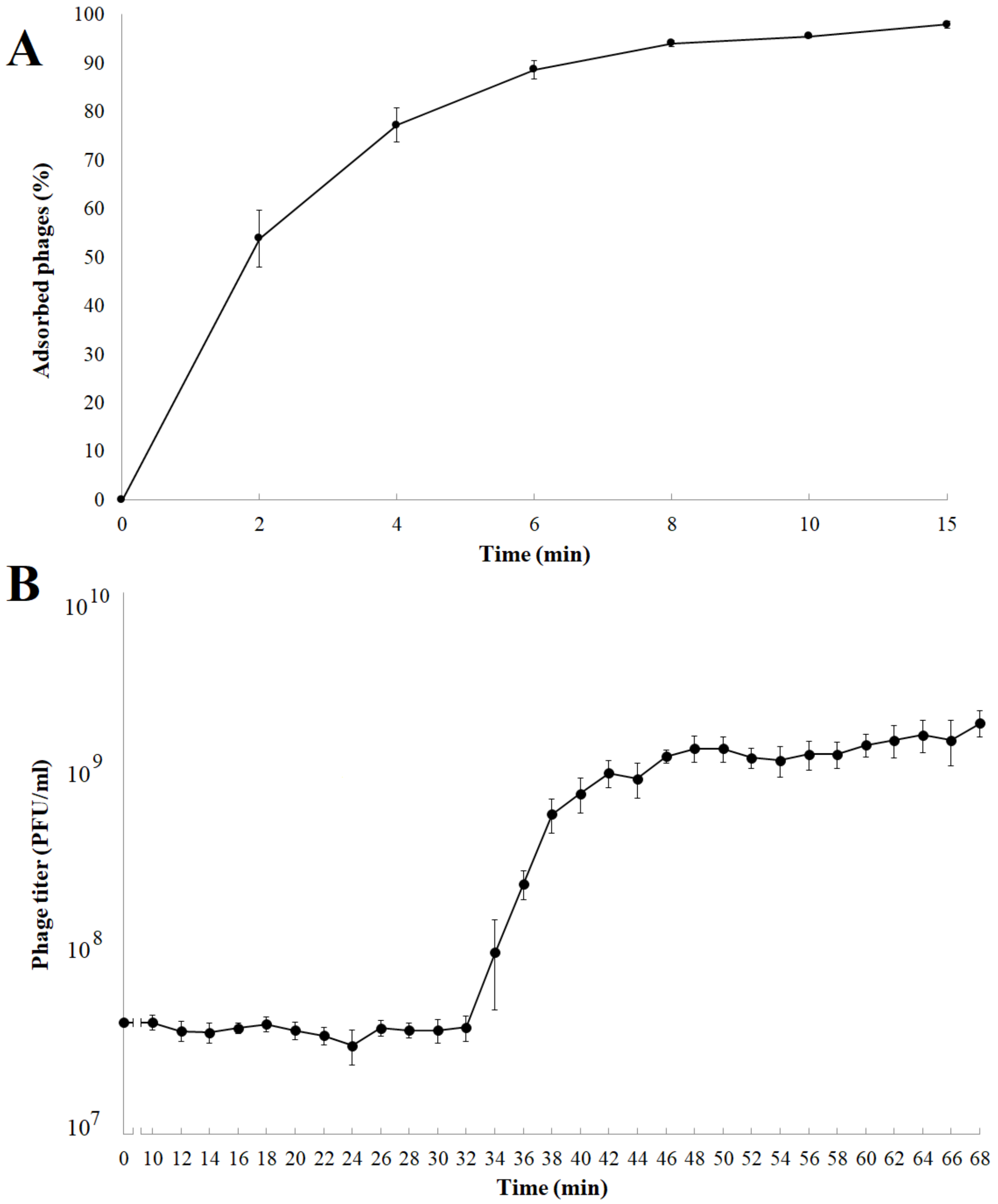

Supplementary Figure 1. (A) Adsorption rate and **(B)** one-step growth curve of phage phiAGO1.3. *S. aureus* strain 80wphwpl was used as phage host. Cultures for the measurements were grown at 37°C. Points on each curve represent average values from three independent experiments.

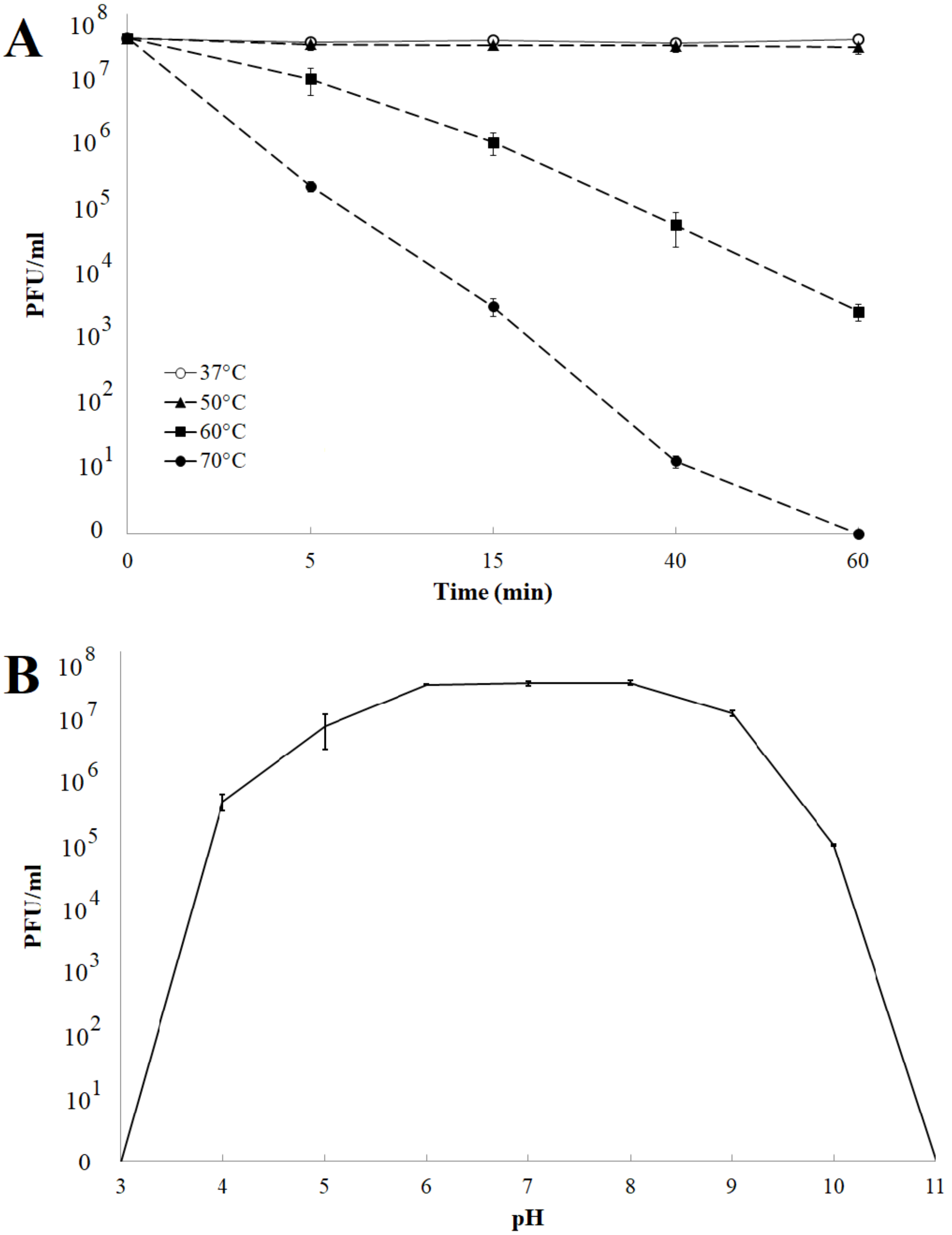

Supplementary Figure 2. Stability of phage phiAGO1.3 at different temperatures **(A)** and at different pHs **(B)**. The influence of pH on phage infectivity was assayed after one hour incubation at given pH. Each point in either curve curve represents the average measurement from three independent experiments.

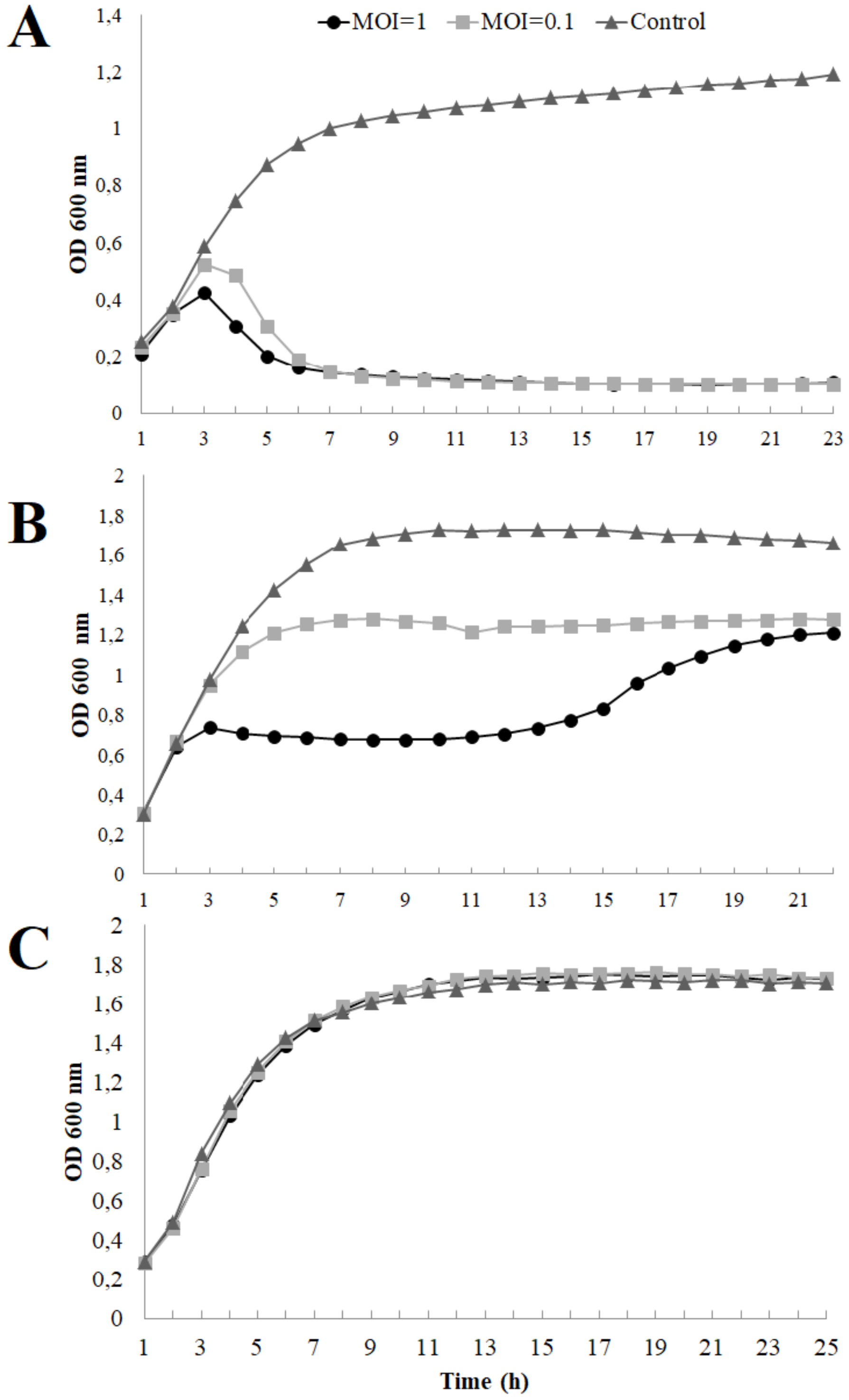

Supplementary Figure 3. Exemplary patterns of optical density changes of *S. aureus* cell cultures of various strains upon addition of phiAGO1.3. **(A)** Complete lysis, strain 3008/05. **(B)** Partial lysis, strain 3009/07. **(C)** Lack of lysis, strain 1330/05.

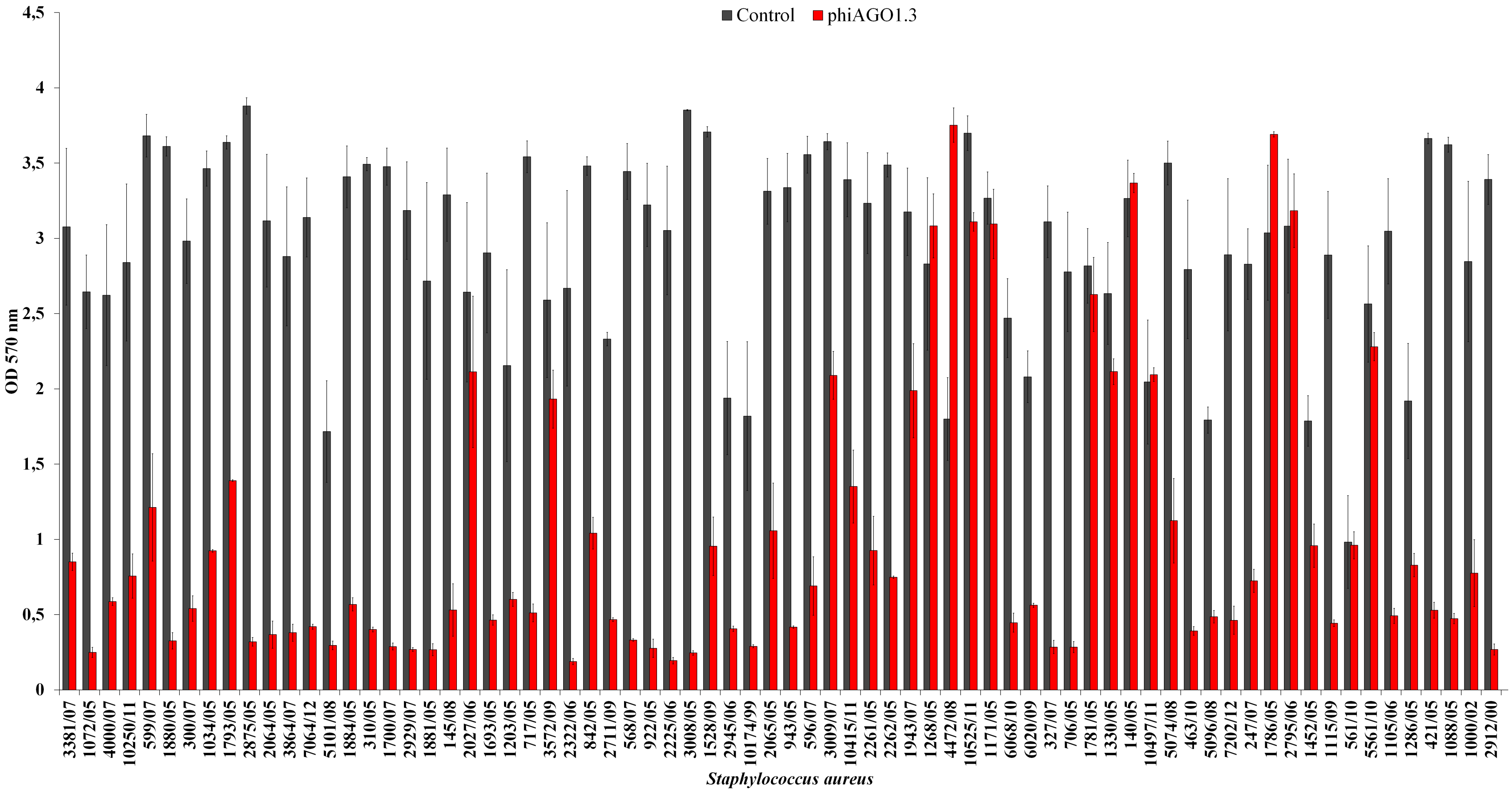

Supplementary Figure 4. Activity of phiAGO1.3 in the biofilm disruption of various *S. aureus* strains.

3 Supplementary References

Adams, M. H. (1959). *Bacteriophages.* New York: Interscience Publishers

Shao, Y., and Wang, I. N. (2008). Bacteriophage adsorption rate and optimal lysis time. *Genetics.* 180, 471-82. doi: 10.1534/genetics.108.090100

Szczepankowska, A. K., Szatraj, K., Sałański, P., Rózga, A., Górecki. R. K., and Bardowski, J. K. (2017). Recombinant *Lactococcus lactis* expressing haemagglutinin from a Polish avian H5N1 isolate and its immunological effect in preliminary animal trials. *Biomed. Res. Int.* 6747482. doi: 10.1155/2017/6747482
